# *Cx3cr1* controls kidney resident macrophage heterogeneity

**DOI:** 10.3389/fimmu.2023.1082078

**Published:** 2023-05-15

**Authors:** Alex Yashchenko, Sarah J. Bland, Cheng J. Song, Ummey Khalecha Bintha Ahmed, Rachel Sharp, Isabella G. Darby, Audrey M. Cordova, Morgan E. Smith, Jeremie M. Lever, Zhang Li, Ernald J. Aloria, Shuja Khan, Bibi Maryam, Shanrun Liu, Michael R. Crowley, Kenneth L. Jones, Lauren A. Zenewicz, James F. George, Michal Mrug, David K. Crossman, Katharina Hopp, Stavros Stavrakis, Mary B. Humphrey, Florent Ginhoux, Kurt A. Zimmerman

**Affiliations:** ^1^ Department of Internal Medicine, Division of Nephrology, University of Oklahoma Health Sciences Center, Oklahoma City, OK, United States; ^2^ Department of Cell, Developmental, and Integrative Biology, University of Alabama at Birmingham, Birmingham, AL, United States; ^3^ Department of Cell Biology, University of Oklahoma Health Sciences Center, Oklahoma City, OK, United States; ^4^ Department of Medicine, Division of Nephrology, University of Alabama at Birmingham, Birmingham, AL, United States; ^5^ Department of Surgery, Division of Cardiothoracic Surgery, University of Alabama at Birmingham, Birmingham, AL, United States; ^6^ Department of Biochemistry and Molecular Genetics, University of Alabama at Birmingham, Birmingham, AL, United States; ^7^ Department of Genetics, University of Alabama at Birmingham, Birmingham, AL, United States; ^8^ Department of Microbiology and Immunology, University of Oklahoma Health Sciences Center, Oklahoma City, OK, United States; ^9^ Department of Veterans Affairs Medical Center, Birmingham, AL, United States; ^10^ Department of Medicine, Division of Renal Diseases and Hypertension, Polycystic Kidney Disease Program, University of Colorado Anschutz Medical Campus, Aurora, CO, United States; ^11^ Department of Internal Medicine, Division of Cardiovascular Diseases, University of Oklahoma Health Sciences Center, Oklahoma City, OK, United States; ^12^ Department of Internal Medicine, Division of Rheumatology, Immunology, and Allergy, University of Oklahoma Health Sciences Center, Oklahoma City, OK, United States; ^13^ Department of Veterans Affairs Medical Center, Oklahoma City, OK, United States; ^14^ Singapore Immunology Network (SIgN), Agency for Science, Technology and Research (A*STAR), 8A Biomedical Grove, Immunos, Singapore, Singapore

**Keywords:** macrophage heterogeneity, kidney macrophages, CX3CR1, CCR2, fate mapping, single cell RNA sequencing (scRNAseq), cystic kidney disease, parabiosis

## Abstract

Kidney macrophages are comprised of both monocyte-derived and tissue resident populations; however, the heterogeneity of kidney macrophages and factors that regulate their heterogeneity are poorly understood. Herein, we performed single cell RNA sequencing (scRNAseq), fate mapping, and parabiosis to define the cellular heterogeneity of kidney macrophages in healthy mice. Our data indicate that healthy mouse kidneys contain four major subsets of monocytes and two major subsets of kidney resident macrophages (KRM) including a population with enriched *Ccr2* expression, suggesting monocyte origin. Surprisingly, fate mapping data using the newly developed *Ms4a3^Cre^ Rosa Stop^f/f^ TdT* model indicate that less than 50% of *Ccr2^+^* KRM are derived from Ly6c^hi^ monocytes. Instead, we find that *Ccr2* expression in KRM reflects their spatial distribution as this cell population is almost exclusively found in the kidney cortex. We also identified *Cx3cr1* as a gene that governs cortex specific accumulation of *Ccr2^+^* KRM and show that loss of *Ccr2^+^* KRM reduces the severity of cystic kidney disease in a mouse model where cysts are mainly localized to the kidney cortex. Collectively, our data indicate that *Cx3cr1* regulates KRM heterogeneity and niche-specific disease progression.

## Introduction

It has recently become appreciated that macrophages in most tissues arise from two distinct origins. For example, monocyte-derived macrophages originate from adult hematopoietic stems cells, express high levels of CD11b and low levels of F4/80 (CD11b^hi^, F4/80^lo^), are dependent on the transcription factor *Myb*, and are rapidly turned over through bone marrow monocyte precursors (1). In contrast, tissue resident macrophages originate from embryonic precursors, express low levels of CD11b and high levels of F4/80 (CD11b^lo^, F4/80^hi^), are *Myb* independent, and minimally rely on bone marrow monocytes for maintenance (1). Further adding to the complexity, it is now appreciated that resident macrophages can be derived from multiple sources including *Myb-*independent yolk-sac progenitors, erythro-myeloid progenitors (EMPs), fetal liver monocytes, and adult hematopoietic stem cell (HSC)-derived Ly6c^hi^ monocytes (2–4). These data, in addition to data showing that unique macrophage populations occupy distinct niches across tissues (5–7), led to the concept that resident macrophage diversity is driven by a combination of ontological origin, time spent in the tissue, niche-specific cues (i.e. localization), as well as exposure to environmental pathogens or inflammatory stimuli (8). Previous data suggest that ontological origin and spatial orientation may influence kidney resident macrophage (KRM) heterogeneity (9, 10); however, a thorough analysis of variables that dictate this heterogeneity has not been performed to date.

The ability of HSC-derived Ly6c^hi^ monocytes to differentiate into resident macrophages is well-established in the gut and skin where newly arrived monocytes upregulate macrophage markers upon entering the tissue (11–13). Ly6c^hi^ monocytes also have the ability to differentiate into macrophages during inflammation and tissue injury in the gut(13), skin(12, 14), and serous cavity(13). With the development of novel lineage tracing tools with increased specificity for monocyte-derived progeny, it is now possible to track these cells as they enter the tissue and differentiate into resident macrophages (15). In fact, recent studies using *Ms4a3^Cre^ Rosa Stop^f/f^ TdT* mice, which can be used to fate map Ly6c^hi^ monocytes, showed that a large number of resident macrophage populations received a significant contribution from Ly6c^hi^ monocytes, with the exception of the liver, epidermis, and brain (15). Previous data also indicate that the majority of recently arrived, Ly6c^hi^ monocyte-derived resident macrophages express the gene *Ccr2*, which is required for emigration of Ly6c^hi^ monocytes out of the bone marrow (16).

*Cx3cr1* is a G-protein coupled receptor that serves as a chemoattractant and survival signal for monocytes, macrophages, dendritic cells, and T cells (17). In the kidney, *Cx3cr1* regulates monocyte and macrophage accumulation following ischemia reperfusion injury (IRI); loss of *Cx3cr1* is associated with improved kidney function and reduced macrophage accumulation following IRI (18, 19). The idea that *Cx3cr1* promotes kidney disease is supported by studies from a hypertensive mouse model showing reduced macrophage infiltration and interstitial fibrosis in *Cx3cr1* deficient kidneys compared to control kidneys (20). In contrast, *Cx3cr1* plays a protective role in a unilateral ureteral obstruction (UUO) model of chronic kidney disease although loss of *Cx3cr1* once again reduced the number of kidney macrophages and dendritic cells (21). Collectively, these data indicate that *Cx3cr1* regulates macrophage and dendritic cell accumulation in the adult kidney; however, the role of *Cx3cr1*-dependent phagocytes in kidney disease is still controversial.

Chronic kidney disease (CKD) is a leading public health concern affecting ~15% of the adult population worldwide (22). Patients with CKD experience gradual loss of kidney function eventually resulting in end stage kidney disease (ESKD). Approximately 5-10% of all patients with ESKD have autosomal dominant polycystic kidney disease (ADPKD), making it the most common genetically inherited cause of ESKD (23, 24). ADPKD is caused by mutations in cilia related genes (*PKD1, PKD2*) and results in large fluid filled cysts scattered throughout the kidney. Mice with a mutation in *Ift88*, which is required for proper cilia formation (25), represent a ciliopathy mouse model of kidney cystic disease. Conditional deletion of *Ift88* in adult mice leads to slowly progressing cystic disease, which can be greatly accelerated by kidney injury (26). Of note, ischemia reperfusion (IR) injury to conditional *Ift88* mice results in rapid cyst expansion in the kidney cortex (10, 26), mainly due to the fact that ischemic injury predominantly affects the proximal tubular segments of the nephron (27).

To analyze the diversity of monocytes and tissue resident macrophages in the kidney, we performed scRNAseq on CD11b and F4/80 positive cells isolated from the kidney of 6-8-week-old control mice. Our scRNAseq data identified four subsets of infiltrating monocytes (IMs) and two subsets of kidney resident macrophages (KRM) including one KRM subset with enriched expression of the chemokine receptor *Ccr2.* Pseudotime analysis of scRNAseq data suggest that *Ccr2^+^* KRM are derived from Ly6c^hi^ monocytes. Quite surprisingly, fate mapping data indicate that *Ccr2* expression is only partially indicative of monocytic origin, but instead informs spatial localization as *Ccr2^+^* KRM are almost exclusively found in the kidney cortex. Further, we found that *Cx3cr1* regulated cortex specific accumulation of *Ccr2^+^* KRM as loss of *Cx3cr1* reduced the number of cortical *Ccr2^+^* KRM and delayed cyst progression in a cortex-specific model of cystic kidney disease. Thus, our data indicate that KRM heterogeneity is driven by spatial distribution and identify a gene whose is expression is required for maintenance of the aforementioned spatial distribution.

## Results

### scRNAseq reveals the cellular diversity of monocytes and tissue resident macrophages in the kidney

**Figure 1:**
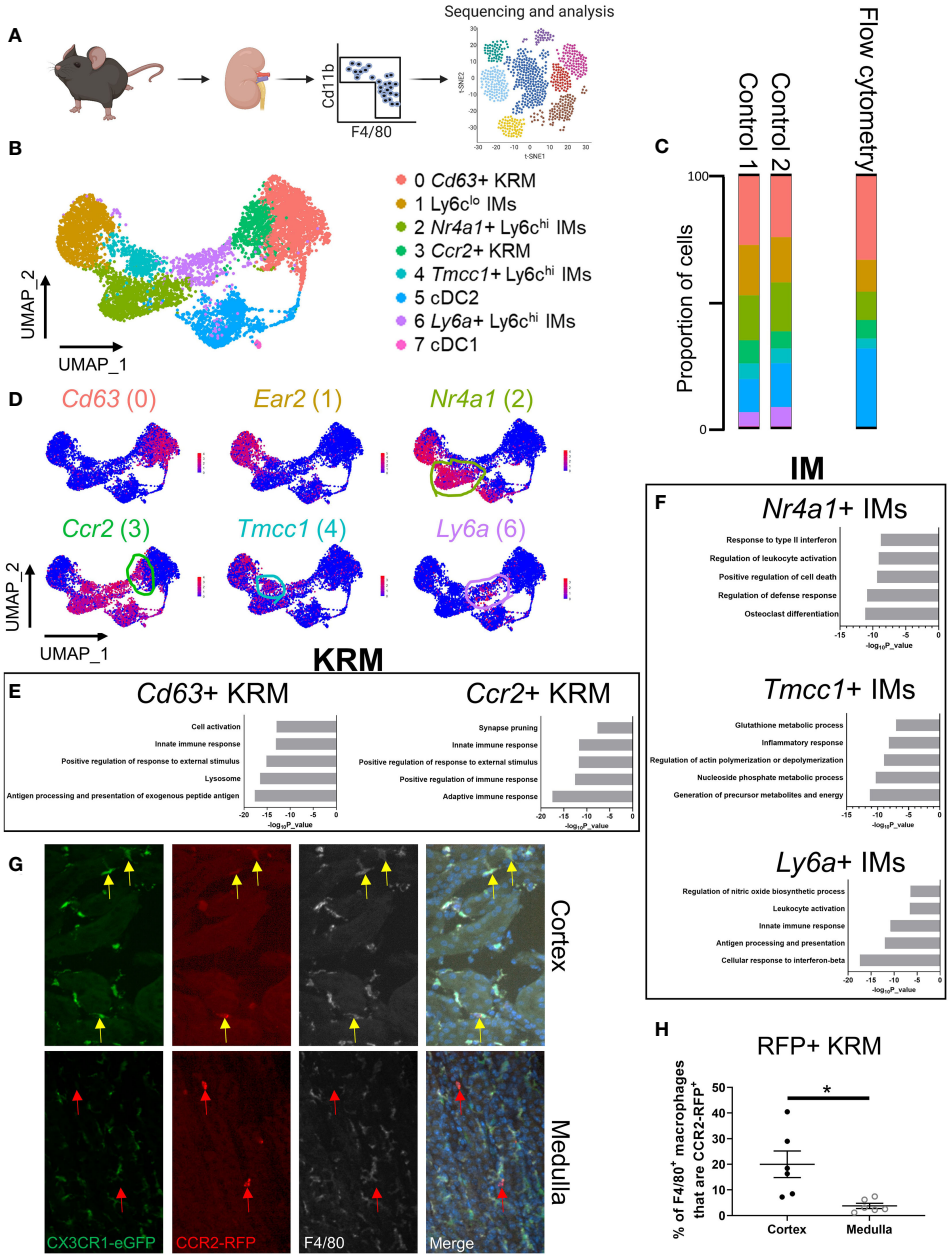
scRNAseq reveals the cellular diversity of monocytes and tissue resident macrophages in the kidney. **(A)** Schematic of the strategy used to perform scRNAseq on CD11b^+^ and F4/80^+^ immune cells isolated from control mice. The control mice for this study were littermates of the *Cx3cr1* ^gfp/gfp^ knockout mice shown in **Figure 3**. **(B)** UMAP of myeloid cells identified via single cell RNA sequencing. **(C)** Quantification of cluster abundance in scRNAseq and flow cytometry data. For flow cytometry, we used surrogate markers to identify *Nr4a1^+^* IMs (UPAR), *Tmcc1^+^* IMs (LRP1), and *Ccr2^+^* KRM (Clec12a). N=5 mice. **(D)** Feature plot showing top DEGs in each cell cluster. **(E, F)** Metascape pathway analysis of genes that were significantly enriched (adjusted p value < 0.05) in **(E)** KRM or **(F)** IM subsets. **(G)** Representative confocal microscopy images of kidneys harvested from *Ccr2* ^rfp/wt^ *Cx3cr1* ^gfp/wt^ mice that were stained with a pan-macrophage marker, F4/80. Yellow arrows depict *Ccr2^+^* KRM while red arrows depict *Ccr2^+^* cells that lacked *Cx3cr1* and F4/80 expression. Images from both the cortex and medulla are shown. Images were taken with a 20X objective. N=3 mice. **(H)** Quantification of RFP, F4/80 double positive macrophages in the cortex and medulla. T-test. *P< 0.05.

To examine the diversity of kidney macrophages, we performed single cell RNA sequencing (scRNAseq) on all CD11b and F4/80 positive cells that were isolated from 6-8-week-old control mice (**Figure 1A**). The gating strategy used for sorting CD11b and F4/80 positive cells from the kidney is shown in **Supplemental Figure 1A**. After quality control and removal of low-quality cells (see methods), we analyzed 6,973 cells from two 6-8-week-old control mice (biological duplicates). Using top differentially expressed genes and previously published literature (28–30), we identified and manually annotated the 8 clusters of cells: Cluster 0 (*Cd63^+^* KRM) expressed classical KRM genes (*C1qa, Cd81*) as well as *Cd63* and *Pf4*; Cluster 1 (Ly6c^lo^ infiltrating monocytes [IMs]) expressed *Ear2* and *Cebpb*; Cluster 2 (*Nr4a1^+^* Ly6c^hi^ IMs) expressed classical monocyte markers (*Ly6c2, Plac8, Chil3*) as well as *Nr4a1* and *Plaur*; Cluster 3 (*Ccr2^+^* KRM) expressed classical KRM genes as well as *Ccr2*, *Mmp12*, and *Clec12a*; Cluster 4 (*Tmcc1^+^* Ly6c^hi^ IMs) expressed classical monocyte markers as well as *Tmcc1* and *Lrp1*; Cluster 5 (cDC2) expressed *Cd209a* and *Clec10a*; Cluster 6 (*Ly6a^+^* Ly6c^hi^ IMs) expressed classical monocyte markers as well as *Ly6a*; Cluster 7 (cDC1) expressed *Xcr1* and *Snx22* (**Figures 1B–D**; **Supplemental Figure 1B**; **Supplemental File 1**). Of note, there was a significant number of type 2 dendritic cells located within the CD11b and F4/80 positive gates, something that is not well established in the kidney. We also confirmed the lack of neutrophils in our scRNAseq data using the mouse neutrophil marker *S100a8* (29) as *Ly6g* transcripts were not detected in our data set (**Supplemental Figure 1C**).

To confirm the cellular diversity identified *via* scRNAseq, we performed flow cytometry on immune cells isolated from kidneys of 6 to 8-week-old male and female wild type mice. Ly6c^hi^ monocyte subtypes were identified *via* surrogate markers found in scRNAseq data (UPAR [*Plaur*] for *Nr4a1^+^* IMs; LRP1 for *Tmcc1^+^* IMs). We chose to use surrogate markers to identify *Nr4a1* ^+^ and *Tmcc1* ^+^ IMs as Nr4a1 and Tmcc1 are intracellularly localized proteins and there is a lack of a well-established, commercially available antibody against Tmcc1. Surrogate markers were chosen based on the following criteria: 1) cell surface localization, 2) commercial availability, 3) strong enrichment in cluster of interest (**Figure 1D**; **Supplemental Figure 1B**). *Ccr2^+^* KRM were identified using the C-Type Lectin Domain Family 12 Member A (Clec12a) surrogate marker as we were unable to identify *bona fide* CCR2^+^ KRM (using a CCR2 antibody; either clone:SA203G11 or clone:475301) *via* flow cytometry (**Supplemental Figures 2A, B**). Flow cytometry data confirmed the presence and proportion of each subset of IMs, dendritic cells, and KRM in healthy mouse kidneys (**Figure 1C**). We also performed a head to head comparison, using both flow cytometry and scRNAseq, of our new gating strategy and a commonly accepted gating strategy for monocytes and tissue resident macrophages to show how our approach captured previously unappreciated heterogeneity within the kidney macrophage niche. Based on the data, the major benefit of using scRNAseq to unbiasedly assess macrophages in the kidney was an increased appreciation for heterogeneity within the Ly6c^hi^ IM and KRM compartment (**Supplemental Figures 2C, D**; **Supplemental Figures 3A, B**). Of interest, we found both *Nr4a1^+^* and *Tmcc1^+^* Ly6c^hi^ monocytes in the blood; however, we were unable to detect *Ly6a^+^* Ly6c^hi^ monocytes (SCA-1^+^) in the blood of healthy mice, similar to recent reports (**Supplemental Figures 4A–C**) (31, 32).

To further validate our scRNAseq findings, we harvested and analyzed KRM isolated from *Ccr2* ^rfp/wt^ *Cx3cr1* ^gfp/wt^ knock-in mice at 6-8 weeks of age. In these mice, insertion of RFP (*Ccr2*) or GFP (*Cx3cr1*) into a single *Ccr2* or *Cx3cr1* allele results in a phenotypically normal mouse with RFP or GFP reporter activity, respectively; insertion of RFP or GFP into both alleles results in a knockout mouse, which still retains reporter activity (33, 34). The *Cx3cr1*-GFP knock-in reporter was included as it can be used to identify KRM (35). In agreement with recent data (35), we found that the majority of KRM express *Cx3cr1* although *Cx3cr1* was also expressed to a similar degree in Ly6c^hi^ IMs (**Supplemental Figure 5A**). Analysis of RFP expression in *Ccr2* ^rfp/wt^ *Cx3cr1* ^gfp/wt^ mice *via* flow cytometry revealed that ~20% of KRM express *Ccr2* (RFP^+^), in line with our scRNAseq and flow cytometry data using the Clec12a surrogate marker (**Supplemental Figure 5B**). Expression of *Ccr2* in IMs and *Ccr2^+^* KRM, but not *Ccr2^-^* KRM, was verified by fluorescence-activated cell sorting (FACS) and qRT-PCR (**Supplemental Figure 5C**).

To better explore possible functions of monocytes and tissue resident macrophages identified in **Figure 1A**, we performed a pathway analysis on genes that were enriched in each cluster of cells compared to all other cells in the UMAP. Pathway analysis data indicate that *Cd63^+^* KRM have an enrichment of genes associated with antigen processing and presentation (*H2-DMb1, H2-Dma, H2-Eb1*), the lysosome (*Ctsh, Ctsc, Ctsb*), and cell activation (*Zfp36l1, H2-DM1, Mafb*) while *Ccr2^+^* KRM have an enrichment of genes associated with adaptive immune response (*H2-DMb1, H2-Aa, H2-Dma*), positive regulation of immune response (*Ighm, Lat2, Cd79b*), and positive regulation of response to external stimulus (*Unc93b1, Scimp, Cd74*; **Figure 1E**). For IM subsets, *Nr4a1^+^* IMs had an enrichment of genes associated with osteoclast differentiation (*Il1b, Tnfrsf1a, Fos*), regulation of defense response (*Il1b, Tnfrsf1a, Ninj1*), and positive regulation of cell death (*Pmaip1, Tnfrsf1a, Lgals1*); *Tmcc1^+^* IMs had an enrichment of genes associated with generation of precursor metabolites and energy (*Cox8a, Cox5b, Cox4i1*), nucleoside phosphate metabolic process (*Ndufb2, Atp5h, Atp5c1*), and glutathione metabolic process (*Gpx1, Arl6ip5, Mgst1*); *Ly6a^+^* IMs had an enrichment of genes associated with cellular response to interferon-beta (*Ifi203, Ifi211, Ifi213*), antigen processing and presentation (*H2-DMb1, H2-Dma, Fcgr2b*), and innate immune response (*Klrk1, Irf8, Igtp*; **Figure 1F**).

We also identified and analyzed genes that were unique to each IM or KRM subset compared to other IM or KRM clusters, respectively. For example, when we did a head to head comparison of *Cd63^+^* and *Ccr2^+^* KRM, we found that *Cd63^+^* KRM had an enrichment of genes associated with the adaptive immune response, regulation of mononuclear cell migration, and positive regulation of endocytosis while *Ccr2^+^* KRM had an enrichment of genes associated with cytoplasmic translocation, ribosome biogenesis, and translation elongation (**Supplemental Figure 5D**). Head to head comparison of IM subsets revealed that *Nr4a1^+^* IMs had an enrichment of genes associated with the inflammatory response, regulation of cell activation, and positive regulation of cell death; *Tmcc1^+^* IMs had an enrichment of genes associated with the inflammatory response, chemotaxis, and nucleoside phosphate metabolic process; *Ly6a^+^* IMs had an enrichment of genes associated with antigen processing and presentation, innate immune response, and positive regulation of immune response (**Supplemental Figure 5E**). Thus, based on our collective data, we propose that in steady state kidneys, both subsets of KRM are involved in the inflammatory response and activating the adaptive immune system; however, our data suggest that *Cd63^+^* KRM may be more involved in endocytosis/phagocytosis and mononuclear cell recruitment whereas *Ccr2^+^* KRM may be the more active cytokine producers (due to enrichment of genes associated with transcription and translation). Our data also strongly suggest that *Ly6a^+^* IMs are a transitionary monocyte due to enriched expression of genes associated with classical macrophage function such as antigen processing and presentation. Finally, we propose that *Nr4a1^+^* IMs have a function that is typically associated with inflammatory monocytes while *Tmcc1^+^* IMs appear to be highly metabolic cells; the function of these cells in steady state is not clear.

To determine the spatial distribution of *Ccr2* ^+^ KRM, we performed confocal microscopy on kidney sections harvested from dual label (*Ccr2* ^rfp/wt^, *Cx3cr1* ^gfp/wt^) mice that had been stained with the pan-macrophage marker F4/80. Our data indicate that approximately 20% of F4/80^+^, GFP^+^ macrophages in the kidney cortex express *Ccr2* (yellow arrows) whereas less than 5% of medullary macrophages express *Ccr2* (**Figures 1G, H**; **Supplemental Figure 5F**). Of interest, a large proportion of RFP*^+^* cells in the medulla lacked expression of *Cx3cr1* and F4/80 (red arrows), suggesting that these may be bone marrow derived dendritic cells (**Figure 1G**; **Supplemental Figure 5F**), which are known to express *Ccr2* (36). Unfortunately, we were unable to verify CD63^+^ KRM localization *via* IF due to poor antibody efficacy. Collectively, the data indicate that *Ccr2^+^* KRM occupy a unique niche in the kidney cortex where they are primed to produce cytokines in response to challenge.

### *Ccr2^+^* KRM are only partially derived from bone marrow monocytes

**Figure 2:**
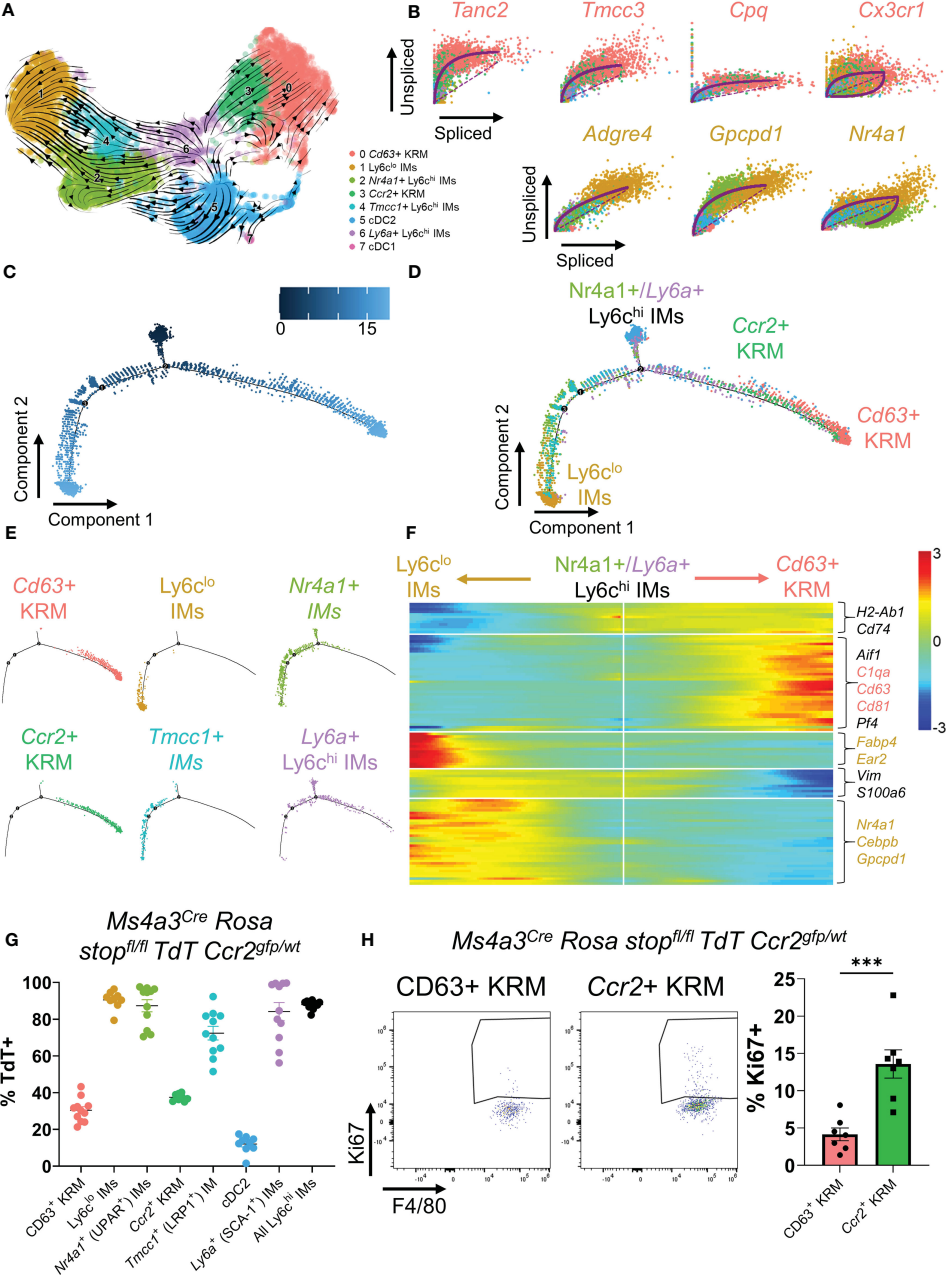
*Ccr2^+^* KRM are partially derived from Ly6c^hi^ monocytes. **(A)** RNA velocity plot of myeloid cells from **Figure 1**. **(B)** List of the top predicted genes that are upregulated as IMs differentiate into *Ccr2^+^/Cd63^+^* KRM (top) or Ly6c^lo^ IMs (bottom). Monocle data showing **(C)** pseudotime and **(D)** cluster composition of myeloid cells from **Figure 1**. **(E)** Branched Monocle2 data showing individual clusters of cells. **(F)** Heatmap showing a list of genes that change as a function of pseudotime (Monocle). **(G)** Quantification of the proportion of each IM and KRM subset that was TdT^+^ in *Ms4a3^Cre^ Rosa stop^fl/fl^ TdT Ccr2* ^gfp/wt^ knock-in fate mapping mice. N=8-11 mice. One-way ANOVA. **(H)** Representative FACS plots and quantification of the percentage of *Ccr2^+^* or *Cd63^+^* KRM that were Ki67 positive. T-test. ***P<0.001.

To test the hypothesis that *Ccr2^+^* KRM are monocyte-derived, similar to what has been reported in the heart (37, 38), we performed pseudotime analysis of single cell data using RNA velocity (scVelo). RNA velocity predicts the directionality of cell differentiation based on the ratio of spliced to unspliced messenger RNA (39). The directionality of arrows infers the direction of cell differentiation due to upregulation of gene expression. RNA velocity data predict that upon entering the kidney, *Nr4a1^+^* and *Tmcc1^+^* IMs upregulate a set of genes to become Ly6c^lo^ IMs, in agreement with literature (**Figure 2A**) (40). The data also suggest that *Ly6a^+^* IMs upregulate a set of genes to become *Ccr2^+^* KRM and, to a certain extent, *Cd63^+^* KRM (**Figure 2A**). Because *Ly6a^+^* monocytes were not detected in the blood (**Supplementals Figure 4A–C**) and express markers of both monocytes and mature macrophages (**Supplemental Figure 1B**), we propose that this cell population is a transitionary monocyte that is derived from *Tmcc1^+^*/*Nr4a1^+^* blood monocytes and gives rise to *Ccr2^+^*/*Cd63^+^* KRM. The hypothesis that *Tmcc1^+^* and *Nr4a1* ^+^ IMs give rise to *Ccr2^+^*/*Cd63^+^* KRM *via* a *Ly6a^+^* IM intermediate was further supported by data from Monocle (**Figures 2C–F**).

We also identified a list of genes that were upregulated as IMs transition to their terminally differentiated states (Ly6c^lo^ IMs or *Ccr2^+^/Cd63^+^* KRM). In agreement with previous data, we identified *Nr4a1* as a gene that was upregulated as *Nr4a1* ^+^/*Tmcc1^+^* IMs differentiated into Ly6c^lo^ IMs (**Figures 2B, F**) (40). Similarly, we found that *Tanc2, Tmcc3, Cpq*, and *Cx3cr1* were all upregulated as *Ly6a^+^* IMs differentiated into *Ccr2^+^* KRM (**Figure 2B**). Collectively, these data suggest that upon entering the kidney, *Nr4a1^+^* and *Tmcc1^+^* monocytes have the ability to differentiate into either Ly6c^lo^ IMs or *Ccr2^+^/Cd63^+^* KRM.

To directly test the hypothesis that *Ccr2^+^* KRM are derived from Ly6c^hi^ monocytes, we crossed the recently developed *Ms4a3^Cre^ Rosa stop^fl/fl^ TdT* mouse (15) with a *Ccr2* ^gfp^ knock-in reporter mouse (41) and analyzed the percentage of cells expressing TdT in each cluster of cells identified *via* scRNAseq in **Figure 1A**. Quite surprisingly, and in contrast to our initial hypothesis, we found that approximately 30-35% of both subsets expressed TdT, although there was no significant difference between the percentage of CD63^+^ and *Ccr2^+^* KRM that expressed TdT (**Figure 2G**). Our data also revealed reduced TdT labelling in *Tmcc1^+^* IMs compared to *Nr4a1^+^* IMs (**Figure 2G**). In agreement with published data (15), we found significant TdT labelling in Ly6c^hi^ IMs and a paucity of TdT labelling in cDC2, verifying the specificity of the model (**Figure 2G**). These data indicate that monocytes give rise to ~30-35% of KRM and that *Ccr2* expression is not a definitive marker for KRM originating from bone marrow derived Ly6c^hi^ monocytes as described for *Ccr2^+^* cardiac resident macrophages (42).

To test if Ly6c^hi^ monocytes were required for maintaining the KRM niche, we quantified the number of *Ccr2^+^* KRM in control (*Ccr2* ^rfp/wt^) and *Ccr2* knockout mice (*Ccr2* ^rfp/rfp^) *via* flow cytometry at 6 to 8 weeks of age. Prior to analyzing KRM number, we first confirmed that *Ccr2* knockout mice had reduced blood and kidney Ly6c^hi^ monocytes (**Supplemental Figures 6A, B**). Analysis of flow cytometry data revealed that loss of *Ccr2* did not significantly reduce *Ccr2^+^* KRM number (**Supplemental Figures 6C, D**); however, when we aged the *Ccr2* knockout mice to ~6 months, we found that loss of *Ccr2* significantly reduced *Ccr2^+^* KRM number (**Supplemental Figure 6E**). We also analyzed the turnover of KRM subsets *via* intracellular stain with Ki67. Our data indicate that *Ccr2^+^* KRM have significantly increased levels of proliferation when compared to CD63*^+^* KRM (**Figure 2H**). Thus, although monocyte input is similar between KRM subsets, it appears that *Ccr2^+^* KRM are proliferating more rapidly and are partially reliant on Ly6c^hi^ monocyte input to maintain the niche.

### *Cx3cr1* is required for accumulation of *Ccr2^+^* KRM in the kidney cortex

**Figure 3:**
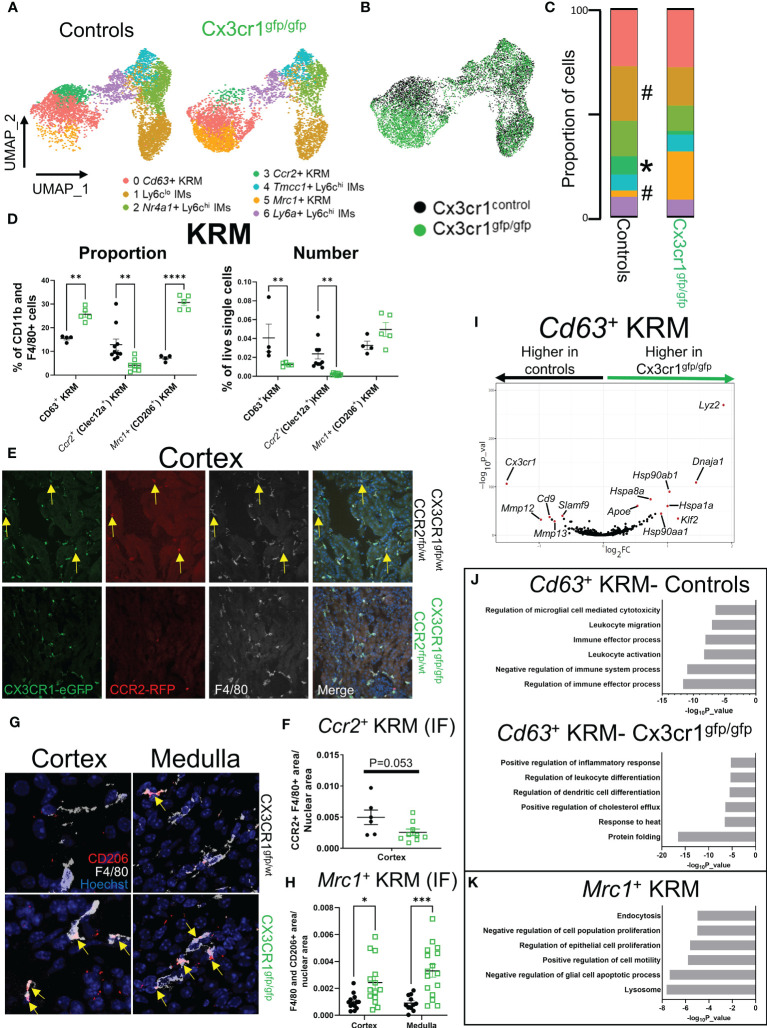
*Cx3cr1* is required for accumulation of *Ccr2^+^* KRM in the kidney cortex. **(A)** UMAP of cell clusters in control (N=2 mice; both female) and *Cx3cr1* ^gfp/gfp^ knockout (N=2 mice; both female) mice. The control group shown in this figure is the same as the data shown in **Figure 1**. **(B)** Composite UMAP of cells from control and *Cx3cr1* ^gfp/gfp^ mice. **(C)** Quantification of cluster composition. T-test. **(D)** Quantification of flow cytometry data analyzing KRM subsets as a percentage of CD11b and F4/80 positive cells or as a percentage of live single cells in the kidney. Two-way ANOVA. **(E)** Representative confocal microscopy images of kidney sections isolated from the cortex of *Cx3cr1* ^gfp/wt^ *Ccr2* ^rfp/wt^ (top) or C*x3cr1* ^gfp/gfp^ *Ccr2* ^rfp/wt^ (bottom) stained with the pan macrophage marker F4/80. Mice were 6-8 weeks of age at harvest. N=2-4 mice per group. **(F)** Quantification of RFP^+^ F4/80^+^ area normalized to total nuclear area in *Cx3cr1* control or knockout mice. Each dot represents an individual image that was quantified from a combined 2-4 mice per group. T-test. **(G)** Representative confocal microscopy images of kidney sections isolated from the cortex (left) or medulla (right) of *Cx3cr1* ^gfp/wt^ (top) or C*x3cr1* ^gfp/gfp^ (bottom) mice stained with the pan macrophage marker F4/80 and CD206. Mice were 6-8 weeks of age at the time of harvest. N=3-4 mice per group. **(H)** Quantification of F4/80^+^ CD206^+^ area normalized to total nuclear area in the cortex or medulla of *Cx3cr1* control or knockout mice. Each dot represents an individual image that was quantified from a combined 3-4 mice per group. Two-way ANOVA. **(I)** Volcano plot showing genes that were enriched in *Cd63^+^* KRM isolated from control or *Cx3cr1* ^gfp/gfp^ kidneys. **(J)** Metascape pathway analysis of genes that were significantly enriched (adjusted p value < 0.05) in *Cd63^+^* KRM isolated from control or *Cx3cr1* ^gfp/gfp^ kidneys. **(K)** Metascape pathway analysis of genes that were significantly enriched (adjusted p value < 0.05) in *Mrc1^+^* KRM compared to *Cd63^+^* KRM. ^#^P <0.1, *P< 0.05, **P< 0.01, ***P< 0.001, ****P< 0.0001.

It has previously been reported that loss of *Cx3cr1* greatly reduces the number of kidney mononuclear phagocytes, particularly in the kidney cortex (18, 21, 43, 44), suggesting that *Cx3cr1* may control the seeding and/or maintenance of cortex localized *Ccr2^+^* KRM. To understand how loss of *Cx3cr1* impacts mononuclear phagocyte subsets in the kidney, we performed scRNAseq on CD11b and F4/80 positive myeloid cells isolated from the kidneys of *Cx3cr1* knockout (*Cx3cr1* ^gfp/gfp^) mice at 6 to 8 weeks of age and compared their transcriptional profile to CD11b and F4/80 positive cells isolated from *Cx3cr1* control mice from **Figure 1**. For these experiments, we sorted an approximately equal number of CD11b and F4/80 positive cells from both genotypes. After removal of contaminating DCs, analysis of cluster composition revealed 7 clusters of cells; 6 of which were identical to the scRNAseq data presented in **Figure 1** (**Figures 3A, B**; **Supplemental Figure 7A**). Interestingly, *Cx3cr1* knockout mice had an additional cluster of cells that was nearly absent in control mice (**Figures 3A, B**). This cluster (Cluster 5; *Mrc1^+^* KRM) expressed classical KRM genes (*C1qa, Cd81*) as well as *Mrc1* (**Supplemental Figure 7A**). Analysis of cluster composition (as a proportion of CD11b and F4/80 positive cells) revealed that *Cx3cr1* knockout mice had a significant reduction in the proportion of *Ccr2^+^* KRM and Ly6c^lo^ IMs as well as a significant increase in the proportion of *Mrc1^+^* KRM compared to control mice (**Figure 3C**). Monocyte subsets were unchanged between *Cx3cr1* control and knockout mice (**Figure 3C**), which was confirmed *via* flow cytometry, both as a proportion of CD11b and F4/80 positive cells and as a frequency of live single cells in the kidney (**Supplemental Figures 8A–C**). We also confirmed that *Cx3cr1* ^gfp/gfp^ mice had reduced *Ccr2^+^* KRM and increased *Mrc1* ^+^ KRM compared to control mice (both as a percentage of CD11b and F4/80 positive cells in the kidney and as a frequency of live single cells in the kidney) by flow cytometry (**Figure 3D**; **Supplemental Figure 8D**) and confocal microscopy (**Figures 3E–H**). Of note, the loss of *Ccr2^+^* KRM was restricted to the kidney cortex while we observed increased *Mrc1^+^* KRM in both the cortex and medulla (**Figures 3E–H**).

To better understand how *Mrc1^+^* KRM were accumulating in *Cx3cr1* ^gfp/gfp^ mice, we performed RNA velocity analysis on the composite UMAP generated from *Cx3cr1* control and knockout mice (**Figure 3B**). RNA velocity analysis indicates that in the absence of *Cx3cr1*, *Mrc1^+^* KRM are likely derived from *Cd63^+^* KRM (**Supplemental Figure 8E**). Whether *Mrc1^+^* KRM accumulate through self-proliferation or monocyte recruitment and engraftment is unknown, although previous data indicate that CD11c^+^ mononuclear phagocytes (MNPs) from *Cx3cr1* ^gfp/gfp^ mice proliferate at a greater rate than CD11c^+^ MNPs from control mice (21). We also analyzed which cells express the only known ligand for CX3CR1*, Cx3cl1*, using previously published scRNAseq data (45). These data indicate that the majority of *Cx3cl1* is expressed in the distal tubule, macula densa, and cells of the deep inner medullary collecting duct (**Supplemental Figure 8F**).

Next, we compared how loss of *Cx3cr1* affected gene expression in *Cd63^+^* KRM (**Figure 3I**), a cluster whose abundance was minimally impacted by loss of *Cx3cr1* (**Figure 3C**). This analysis revealed that *Cd63^+^* KRM from control mice had an enrichment of genes associated with regulation of immune effector process (*Scimp1, Lacc1, Cd86*), leukocyte activation (*Cdk6, Mafb, Spi1*), and leukocyte migration (*Cx3cr1, C5ar1, Ccl12*; **Figures 3I, J**). In contrast, *Cd63^+^* KRM from *Cx3cr1* knockout mice had an enrichment of genes associated with protein folding (*Hspa1a, Hspa1b, Hspa8*), response to heat (*Hsp90aa1, Hsp90ab1, Nfkbia*), and immune cell differentiation (*Tmem176b, Cebpb, Tmem176a, H2-Dma, Cd83, Hsp90aa1*; **Figures 3I, J**). We also confirmed that *Cx3cr1* expression was significantly elevated in control mice compared to *Cx3cr1* ^gfp/gfp^ mice (**Figure 3I**). Overall, these data support the idea that *Cx3cr1* is required for homeostatic maintenance of the KRM niche.

To understand the possible function of *Mrc1^+^* KRM, which were almost exclusively present in *Cx3cr1* ^gfp/gfp^ mice (**Figure 3C**), we performed a pathway analysis on genes enriched in this cluster of cells compared to *Cd63^+^* KRM. The data indicate that *Mrc1^+^* KRM had an enrichment of genes associated with the lysosome (*Ctsb, Ctsl, Comt*), endocytosis (*Cltc, Clta, Ap2a2*), negative regulation of apoptotic cell processes (*Igf1, Gas6, Trem2*), and negative regulation of cell proliferation (*Hmox1, Igf1, Ang*; **Figure 3K**). Of note, several of the genes (i.e. *Trem2, Igf1, Gas6*) and pathways (lysosome, endocytosis, regulation of apoptotic cell processes) enriched in *Mrc1^+^* KRM overlapped with signatures previously assigned to *Trem2^+^* macrophages, a cell type that is believed to restrict disease progression in several tissues including the kidney (46–48).

### Macrophage intrinsic *Cx3cr1* expression is required for KRM niche filling

**Figure 4:**
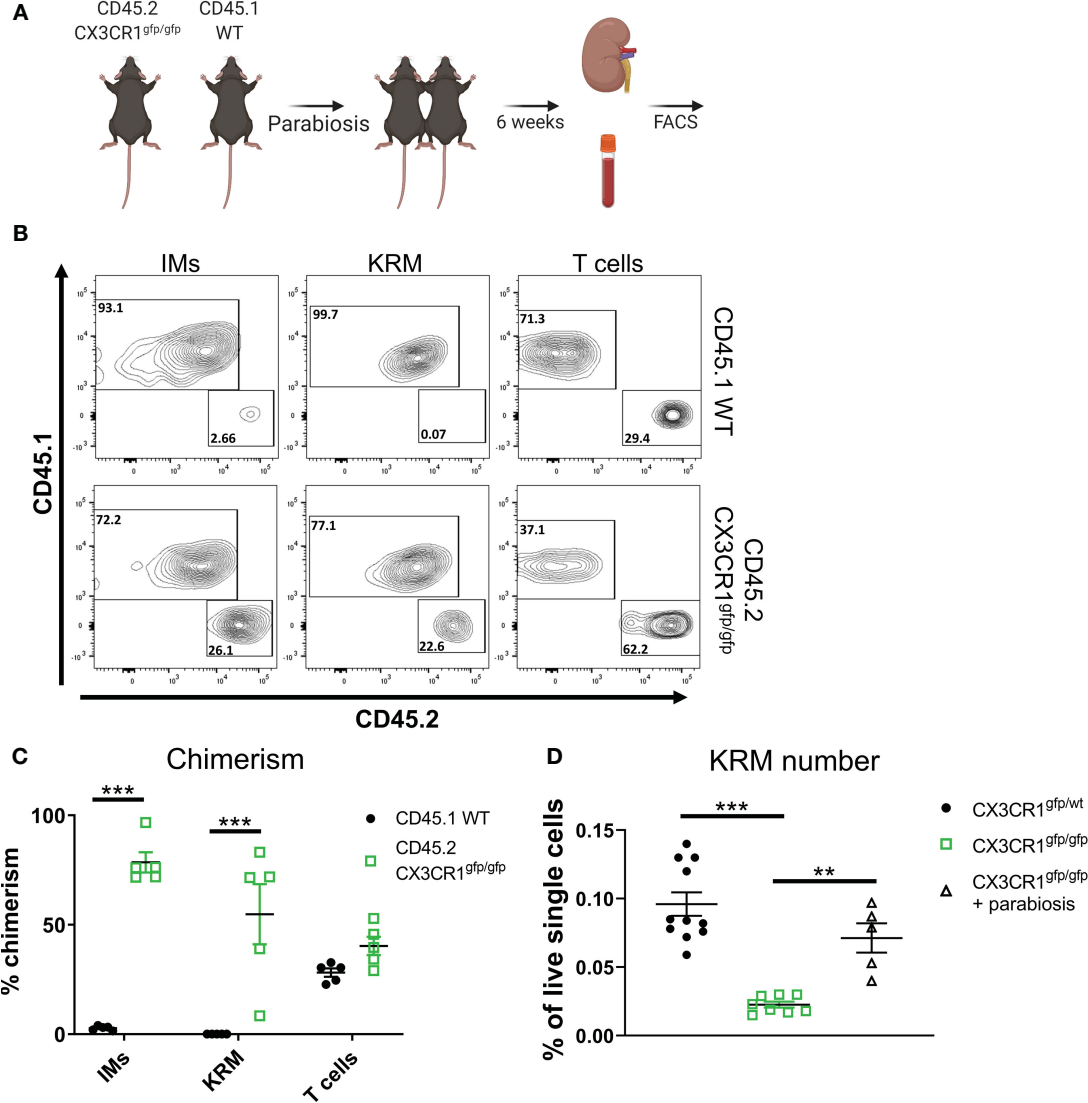
Monocyte intrinsic *Cx3cr1* expression is required for KRM niche filling. **(A)** Schematic of parabiosis experiment. **(B)** Representative FACS plots of IMs, KRM, and T cells from wild type CD45.1 and CD45.2 *Cx3cr1* ^gfp/gfp^ knockout mice 6 weeks post hook-up. **(C, D)** Quantification of **(C)** chimerism and **(D)** KRM number (graphed as a % of live single cells) in wild type CD45.1 and CD45.2 *Cx3cr1* ^gfp/gfp^ knockout mice 6 weeks post parabiosis surgery. Significance was determined by a **(C)** Two-way ANOVA or **(D)** One-way ANOVA. **P< 0.01, ***P< 0.001.

To test if intrinsic (i.e. within monocytes/macrophages) or extrinsic (i.e. within stromal cells) *Cx3cr1* expression is required for maintaining the KRM niche, we employed a parabiosis model in which we joined a CD45.2 *Cx3cr1* ^gfp/gfp^ mouse, which lacks a majority of *Ccr2^+^* KRM, with a congenic CD45.1 wild type mouse *via* parabiosis surgery (**Figure 4A**). Six weeks post parabiosis surgery, we harvested wild type (WT) CD45.1 and CD45.2 *Cx3cr1* ^gfp/gfp^ kidneys and analyzed chimerism percentage in IMs in the blood and kidney as well as KRM. Additionally, the percent chimerism of T cells was analyzed in order to validate an efficient exchange of blood cells between parabiosed animals. Our data indicate that the level of chimerism of T cells in the kidney of CD45.1 WT and CD45.2 *Cx3cr1* ^gfp/gfp^ mice was approximately equal (40%) indicating an efficient exchange of immune cells between mice, similar to previous reports (**Figures 4B, C**) (49). Of note, chimerism of Ly6c^hi^ blood monocytes in both CD45.1 WT and CD45.2 *Cx3cr1* ^gfp/gfp^ mice was ~10%, similar to recent reports (**Supplemental Figure 9A**)(49). Analysis of IMs and KRM in wild type CD45.1 mice revealed minimal chimerism in agreement with other reports(49); in contrast, chimerism of IMs and KRM in CD45.2 *Cx3cr1* ^gfp/gfp^ mice was ~70% and 50%, respectively (**Figures 4B, C**). We also confirmed that in parabiotic mice, wild type infiltrating monocytes filled the kidney cortex niche to compensate for the loss of *Ccr2^+^* KRM that was observed in *Cx3cr1* ^gfp/gfp^ mice (**Figure 4D**). The reduced number of CD45.2 IMs in CD45.2 *Cx3cr1* ^gfp/gfp^ kidneys was not due to reduced numbers of CD45.2 Ly6c^hi^ monocytes in the blood of these mice (**Supplemental Figure 9B**). In fact, our data indicate that loss of *Cx3cr1* did not impact the number of any subset of blood monocytes (**Supplemental Figure 9C**). These data indicate that monocytes, and not stromal cells, require *Cx3cr1* to enter into the kidney and fill an open KRM niche.

### Loss of *Cx3cr1* prevents niche specific cystic kidney disease

**Figure 5:**
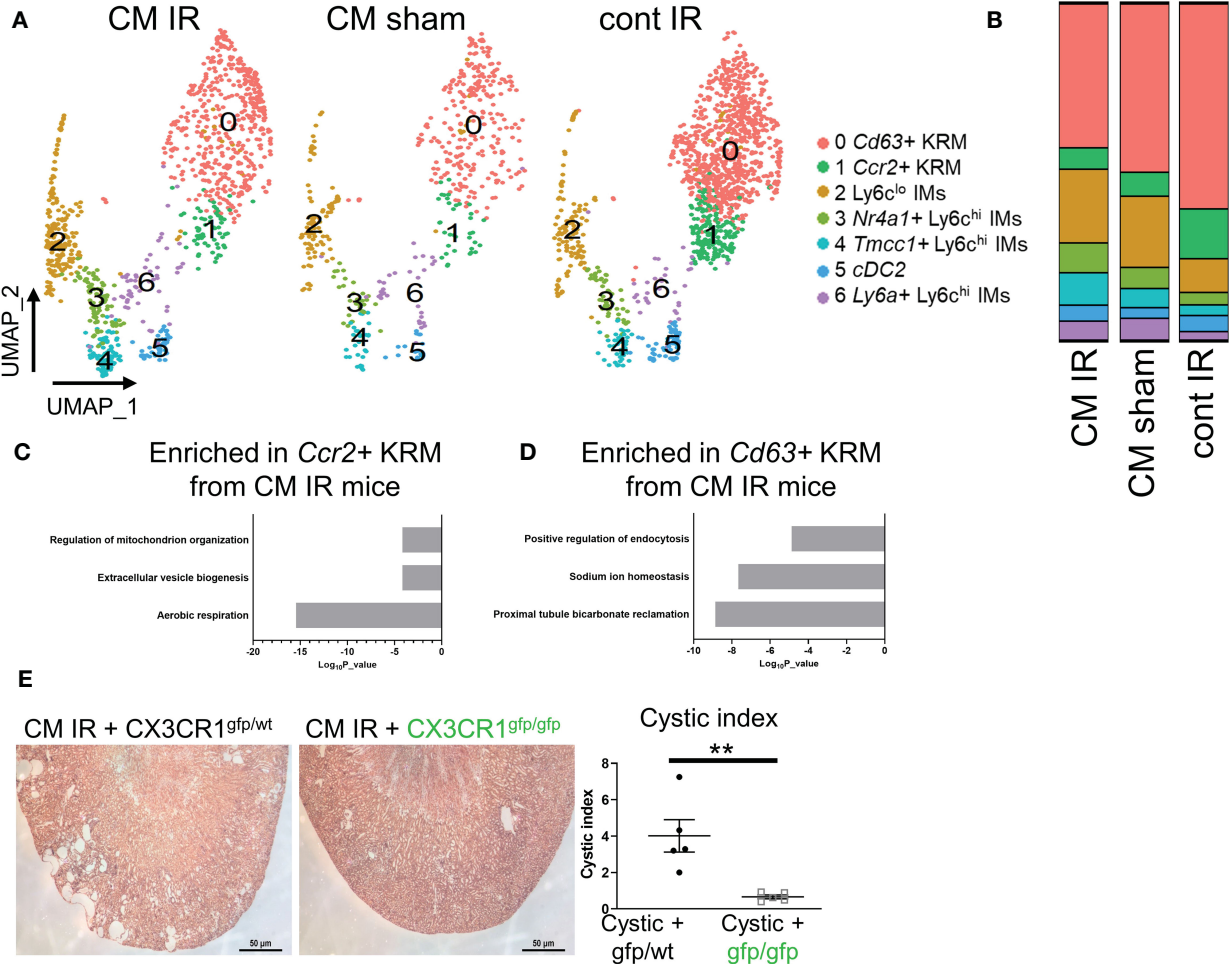
Loss of *Cx3cr1* reduces injury accelerated cystic disease. **(A)** UMAP showing clusters of macrophages and dendritic cells in cystic (CM IR) and control mice (CM sham, cont IR) mice 56 days post IR injury or sham surgery. **(B)** Quantification of cluster composition in biological duplicates. **(C, D)** Pathway analysis of genes that were enriched (adjusted P value < 0.05) in **(C)** *Ccr2^+^* KRM or **(D)** *Cd63^+^* KRM from cystic mice compared to control mice (CM sham and cont IR). **(E)** Representative H&E images and quantification of cystic index in CM IR mice on the *Cx3cr1* ^gfp/wt^ or *Cx3cr1* ^gfp/gfp^ background 56 days post injury. **P< 0.01.

Previous data indicate that IR injury accelerates cystic kidney disease in conditional *Ift88* ^f/f^ mice (26, 50). Our lab has also previously shown that KRM promote cyst progression in this model (10); however, these studies did not address the involvement of specific macrophage subsets in disease progression. To understand how IR injury impacts macrophage subsets in the kidney in the context of cilia dysfunction (i.e. *Ift88* mutation), we re-analyzed our previously published scRNAseq data (GSE193528) and focused on CD11b and F4/80 positive cells isolated from control (cont) or *Ift88* cilia mutant (CM) mice 8 weeks post IR injury (IR) or sham surgery (sham) (50). In agreement with earlier data, we were once again able to identify 3 clusters of Ly6c^hi^ IMs as well as cDC2, *Ccr2^+^* KRM, and *Cd63^+^* KRM (**Figure 5A**). As previously published, we were unable to detect *Mrc1+* KRM in this model(50). Analysis of cluster composition showed no significant difference in the proportion of any cluster of cells when comparing cystic (IR injured cilia mutant mice; CM IR) and control mice (sham operated cilia mutant mice, CM sham; injured control mice, cont IR; **Figure 5B**). We also performed a pathway analysis on genes that were enriched in *Ccr2^+^* and *Cd63^+^* KRM isolated from cystic mice compared to control mice. Of interest, pathway analysis data indicate that *Ccr2^+^* KRM from cystic mice (CM IR) had an enrichment of genes associated with aerobic respiration (*ND1, Ndufs3, Ndufs6*), extracellular vessel biogenesis (*Arrdc1, Cops5, Atp13a2*), and regulation of mitochondrial organization (*Atpif1, Ier3, Chchd10*; **Figure 5C**). In contrast, *Cd63^+^* KRM isolated from cystic mice had an enrichment of genes associated with proximal tubule bicarbonate reclamation (*Atp1b1, Atp1a1, Fxyd2*), sodium ion homeostasis (*Umod, Spp1, Slc12a1*), and positive regulation of endocytosis (*Igf1, Trem2, Lrp1*; **Figure 5D**).

Importantly, IR injury mainly affects the proximal tubule region resulting in rapid cyst expansion in the kidney cortex (26, 27), thus providing us with a model to test the importance of cortex-localized, *Ccr2^+^* KRM in a niche-specific model of disease. To test the hypothesis that *Ccr2^+^* KRM promote cyst progression, we analyzed cyst severity in CM IR mice on the *Cx3cr1* ^gfp/wt^ and *Cx3cr1* ^gfp/gfp^ knockout background eight weeks post injury. In agreement with previous reports (26, 50), our data indicate cysts are mainly found in the cortex in CM IR mice on the *Cx3cr1* control background whereas loss of *Cx3cr1*, and *Ccr2^+^* KRM, significantly reduced cystic disease in the kidney cortex (**Figure 5E**). Thus, our data indicate that *Cx3cr1* controls niche specific *Ccr2^+^* KRM accumulation and, in turn, regulates cyst progression in a model that mainly impacts the kidney cortex.

## Discussion

Recent literature has highlighted the ever-evolving heterogeneity of monocytes and macrophages across and within tissues. In this manuscript, we use scRNAseq to define the cellular diversity of monocytes and tissue resident macrophages in kidneys isolated from healthy mice. We found that healthy mouse kidneys contain two KRM subsets and four infiltrating monocyte (IM) subsets, which we verified *via* flow cytometry. One of the KRM subsets, which had increased *Ccr2* transcripts, was found to preferentially localize to the kidney cortex. We also tested whether this population was exclusively monocyte-derived as recent data indicate that *Ccr2^+^* resident macrophages in the heart are almost completely derived from bone marrow Ly6c^hi^ monocytes (42, 51). Surprisingly, and in contrast to data from the heart, we find that only ~35% of *Ccr2^+^* KRM are monocyte-derived, a value similar to that observed in *Cd63^+^* KRM. Thus, our unbiased approach (scRNAseq) to detail KRM diversity led us to the conclusion that KRM heterogeneity in steady state is driven by spatial distribution. The idea that KRM diversity is driven by spatial distribution is supported by recent data (9, 28).

In this manuscript, we also identified *Cx3cr1* as a gene that was able to control the spatial and transcriptional diversity of KRM as loss of *Cx3cr1* resulted in a significant reduction in the number of cortex localized *Ccr2^+^* KRM. Corresponding to the loss of *Ccr2^+^* KRM, we also found a new subset of KRM (*Mrc1^+^* KRM) that was enriched in kidneys isolated from *Cx3cr1* knockout mice. However, it is unknown how *Mrc1^+^* KRM accumulate in *Cx3cr1* knockout kidneys. Previous data indicate that Cd11c expressing mononuclear phagocytes isolated from *Cx3cr1* knockout kidneys have increased proliferation compared to controls (21). Thus, it is possible that loss of *Ccr2^+^* KRM is sensed by cells in the kidney cortex; these cells then upregulate expression of *Cx3cl1* (CX3CR1 ligand), resulting in migration and proliferation of the remaining *Cd63^+^* KRM to form the *Mrc1^+^* KRM subset. It is also possible that loss of *Ccr2^+^* KRM results in upregulation of chemoattractant cytokines, which facilitate monocyte recruitment and engraftment into *Mrc1^+^* KRM. Similar processes have been described in the liver following loss of Kupffer cells (52–54). It is also interesting to note that the open KRM niche found in *Cx3cr1* ^gfp/gfp^ mice was filled by monocytes from CD45.1 wild type mice in our parabiosis experiment. This occurred despite the fact that CD45.2 *Cx3cr1* ^gfp/gfp^ mice had more CD45.2 monocytes in the blood and had an equal number of IM subsets in the kidney compared to control mice. These data suggest that intrinsic *Cx3cr1* expression in monocytes is dispensable for their entry into the blood; rather, we propose that *Cx3cr1* is specifically required for the engraftment and differentiation of precursor cells (i.e. HSC-derived monocytes, fetal liver monocytes, etc.) into cortex-localized *Ccr2^+^* KRM.

Although our data indicate that Ly6c^hi^ monocytes give rise to ~35% of *Ccr2^+^* KRM and we find that monocytes require *Cx3cr1* to enter into the kidney and engraft into the open KRM niche (parabiosis data), we do not believe that loss of *Cx3cr1* on monocytes is solely responsible for the observed reduction in *Ccr2^+^* KRM in the *Cx3cr1* ^gfp/gfp^ mice. This conclusion is based on our flow cytometry showing that *Ccr2+* KRM number is not impacted in 6-8-week-old *Ccr2* knockout mice. This is important as the studies in *Cx3cr1* ^gfp/gfp^ mice were all done on mice aged 6-8 weeks. Rather, we propose that all precursor cells, including those from the yolk sac and fetal liver, require *Cx3cr1* for seeding the KRM niche in the kidney cortex. The idea that *Cx3cr1* is required for fetal precursors to seed the embryonic KRM niche is supported by the literature (55). Thus, while these data support the idea that the observed reduction of *Ccr2^+^* KRM in 6-8-week-old *Cx3cr1* ^gfp/gfp^ mice was not solely due to defects in monocyte-dependent niche filling, the data also indicate that monocytes are required for maintaining *Ccr2^+^* KRM throughout the animal’s lifespan.

One interesting observation from our scRNAseq studies was the presence of multiple subsets of Ly6c^hi^ IMs in the kidney, which we validated *via* flow cytometry using surrogate markers. Interestingly, when we used these surrogate markers in the blood, we were only able to identify *Nr4a1^+^* monocytes and a minor fraction of *Tmcc1^+^* monocytes. This is an interesting point as our RNA velocity data suggest that *Ly6a^+^* IMs contain the most unspliced mRNA, suggesting they are the most de-differentiated cell type. Based on these collective data, we propose that *Tmcc1* ^+^ and *Nr4a1^+^* Ly6c^hi^ monocytes are the monocyte precursor cells that come from the blood. These cells then enter the kidney and either 1) differentiate to become Ly6c^lo^ IMs by converting unspliced mRNA into mature mRNA or 2) de-differentiate through upregulation of transcription resulting in more unspliced mRNA to become *Ly6a* ^+^ IMs. The intermediate *Ly6a* ^+^ IMs then undergo the standard differentiation process (i.e. cell maturation) resulting in *Ccr2* ^+^ and *Cd63* ^+^ KRM. Also of interest, analysis of TdT expression using the *Ms4a3^Cre^ Rosa stop^fl/fl^ TdT* mouse revealed lower levels of TdT labelling in the *Tmcc1^+^* subset compared to the *Nr4a1^+^* subset. Since the *Ms4a3^Cre^ Rosa stop^fl/fl^ TdT* mouse specifically labels monocytes of the granulocyte monocyte progenitor (GMP) lineage (15), our data suggest that *Tmcc1^+^* monocytes may be descendants of the monocyte-dendritic cell progenitor (MDP) lineage (56) while *Nr4a1^+^* IMs are derived from GMPs. Despite the fact that we were able to identify two distinct monocyte subsets with unique TdT labelling, pseudotime data indicate that both subsets are equally able to differentiate into Ly6c^lo^ IMs. Why multiple subsets of monocytes may exist in the blood and kidney as well as their function and location is unknown and warrants further investigation.

Recent data indicate that three predominant resident macrophage subsets (TLF^+^ [*Timd4, Lyve1, Folr2*], CCR2^+^, MHC2^+^) are present across tissues in mice and humans (51). Although all three populations of macrophages are found in each tissue, the predominance of these subsets differs depending on the tissue analyzed. For example, the liver is mainly comprised of TLF^+^ macrophages while the kidney contains mostly CCR2^+^ and MHC2^+^ macrophages. The data from this manuscript support that finding; however, it should be noted that the proportion of each population that we found in the kidney differed slightly from Dick et al. as we found that ~20-25% of KRM expressed *Ccr2* while the remaining *Cd63^+^* KRM subset (which is equivalent to the MHC2^+^ subset in Dick et al.) comprised the majority of remaining KRM. When we projected a TLF modular score onto our single cell data, we only found a few KRM that had a notable score (not enough to form a cluster), similar to Dick et al. Thus, at least in the kidney, the majority of KRM are either *Ccr2^+^* KRM or *Cd63^+^*/MHC2^+^ KRM.

We were also unable to identify CCR2^+^ KRM using an antibody and standard flow cytometry procedures. This occurred despite our repeated efforts using multiple anti mouse CCR2 antibodies ((BioLegend, Clone: SA203G11) and (BD Biosciences, Clone: 475301)) and standard flow cytometry staining protocols as previously described (37, 38). To ensure the rigor of our experiment, we used the following controls: a CCR2-GFP knock-in reporter (*Ccr2* ^gfp/wt^) and a *Ccr2* knockout animal (*Ccr2* ^gfp/gfp^). If the antibody is effective, we would expect to observe very few KRM that were CCR2^+^ in mice lacking *Ccr2.* Our analysis of the data indicate that loss of *Ccr2* did not affect the number of CCR2^+^ KRM identified using the CCR2 antibody. Thus, we conclude that at least in the kidney, the antibody is not effective in detecting CCR2^+^ KRM.

Our data indicate that loss of *Cx3cr1* significantly reduced cystic kidney disease in our IR injury accelerated model. While we speculate that this rescue is due to loss of *Ccr2^+^* KRM, we recognize that we are also gaining another cluster of cells in this model (*Mrc1^+^* KRM). Thus, it is difficult to determine whether the observed rescue of cystic disease is driven by loss of *Ccr2^+^* KRM or gain of *Mrc1^+^* KRM. We also noticed that *Mrc1^+^* KRM had a very unique transcriptional signature including enriched expression of genes such as *Trem2, Igf1*, and *Gas6*. This is important as this signature has been ascribed to *Trem2^+^* macrophages in other slowly progressing models of disease including Alzheimer’s disease (46, 47). In these reports, the authors showed that loss of *Trem2^+^* macrophages worsened phenotypic outcome indicating a protective role for *Trem2^+^* macrophages in disease (46, 47). The observed rescue of cystic disease in our injury accelerated cystic model is supported by another recent publication from our lab showing that loss of *Cx3cr1* reduced cystic disease in non-injured conditional *Pkd2* mice(57). Thus, it is possible that the observed rescue in both models of cystic disease on the *Cx3cr1* ^gfp/gfp^ background is driven by the accumulation of disease-restricting *Trem2^+^* macrophages. This hypothesis warrants further investigation.

Our studies have several limitations. 1) The control mice that were used for single cell RNA sequencing studies came from *Cx3cr1* ^gfp/wt^ mice, meaning some heterozygous effects may be present in our data set. 2) The mice used for single cell RNA sequencing studies were all female. To address these limitations, we validated our scRNAseq findings in wild type mice *via* flow cytometry, using animals of both sexes, thereby strengthening the cogency of our findings. 3) The mice analyzed in this studied were 6-8 weeks old. It is possible that *Ccr2* expression may more clearly identify resident macrophages of monocyte origin in younger or older mice. 4) We are unable to assess the turnover of monocyte-derived KRM as we used a constitutive Cre recombinase, as opposed to a tamoxifen inducible model, to study the fate of Ly6c^hi^ monocytes in the kidney. We also only analyzed one time point preventing us from dissecting how monocyte input into the KRM niche changes over time.

## Materials and methods

### Mice

Ms4a3^Cre/Cre^ mice were the kind gift of Dr. Florent Ginhoux. *Rosa stop^fl/fl^ TdT* mice (B6.Cg-Gt(ROSA)26Sortm14(CAG-tdTomato)Hze/J; Stock No: 007914), *Ccr2* ^rfp/rfp^ mice (B6.129(Cg)-Ccr2tm2.1Ifc/J; Stock No: 017586), *Ccr2* ^gfp/gfp^ mice (B6(C)-Ccr2tm1.1Cln/J; Stock No: 027619), and *Cx3cr1* ^gfp/gfp^ mice (B6.129P2(Cg)-Cx3cr1tm1Litt/J; Stock No: 005582) were purchased from Jackson Laboratories. CAGG-Cre/Esr1/5Amc/J IFT88^f/f^ (Referred to as cilia mutant mice; CM) C57BL/6J male and female mice were bred in-house. Animals were maintained in Association for Assessment and Accreditation of Laboratory Animal Care International-accredited facilities in accordance with Institutional Animal Care and Use Committee (IACUC) regulations at the University of Alabama at Birmingham (UAB) and University of Oklahoma Health Sciences Center (OUHSC).

### Immunofluorescence microscopy

Following overnight fixation in 4% PFA and cryopreservation in 30% sucrose, 8 µm thick OCT embedded kidney sections were fixed with 4% PFA for 10 minutes, permeabilized with 1% Triton X-100 for 8 min, and incubated in blocking solution (PBS with 1% BSA, 0.3% Triton X-100, 2% (vol/vol) donkey serum, and 0.02% sodium azide) for 30 minutes at room temperature. Sections were incubated in primary antibody overnight at 4°C, washed with PBS, and incubated with the appropriate secondary antibodies in blocking solution for 1 hour at room temperature. The primary antibodies were 1) rat anti-mouse F4/80 (ThermoFisher, Cat no: 14-4801-82, Clone: BM8, diluted 1:200 in blocking solution) and 2) rabbit anti-mouse CD206 (Abcam, Cat no: ab64693, Clone: EPR6868 (B), 1:200 dilution in blocking solution). The secondary antibodies were 1) an Alexa Fluor 647-conjugated anti-rat antibody (Catalog NO: 712-606-153, Jackson ImmunoResearch, 1:250 dilution in PBS) and 2) an Alexa Fluor 568-conjugated donkey anti-rabbit (ThermoFisher, Cat no: A-11011, diluted 1:1000 in blocking solution). Following addition of secondary antibody, nuclei were stained by Hoechst nuclear stain (Sigma-Aldrich) and samples mounted using IMMU-MOUNT (Thermo). All fluorescence images were captured on a Fluoview 1000/IX81 Laser Scanning Confocal microscope (Olympus) with an inverted configuration.

### Flow cytometry

After perfusion of the mouse with PBS, kidneys were minced and digested in 1 ml of RPMI 1640 containing 1 mg/ml collagenase type I (Sigma-Aldrich) and 100 U/ml DNase I (Sigma-Aldrich) for 30 min at 37°C with agitation. Kidney fragments were passed through a 70-µm mesh (Falcon; BD Biosciences) yielding single-cell suspensions. Cells were centrifuged at 1300 rpm (330 X g) for 5 minutes and red blood cells lysed using ACK red blood cell lysis buffer (Quality Biological; 10128-802) at 37°C for 5 minutes. Cells were spun at 1300 rpm (330 X g), resuspended in 1 ml of 1% BSA containing Fc blocking solution (dilution 1:200) and incubated for 30 minutes on ice. Following cell counting with trypan blue, approximately 2 million cells were stained for 30 minutes at room temperature with the following direct conjugated primary antibodies: BV786 rat anti-mouse CD45 (Catalog #: 557659, 30-F11, BD Pharmigen), PerCP-Cy5.5 rat anti-mouse CD63 (Catalog #: 143911, Biolegend), APC rat anti-mouse Clec12a (Catalog #: 143405, Biolegend), FITC rat anti-mouse CD206 (Catalog #:141704, Biolegend), PE-Cy5 Armenian hamster anti-mouse CD11c (Catalog #: 117316, Biolegend), Alexa Fluor 488 rat anti-mouse UPAR (Catalog #: FAB531G, R&D Biosystems), Alexa Fluor 647 mouse anti-mouse LRP1 (Catalog #: NB100-64808, Novus Biologicals), PerCP-Cy5.5 rat anti-mouse Ly-6A/E (SCA-1; Catalog #: 45-5981-82, ThermoFisher), eFluor^®^450 rat anti-mouse F4/80 (Catalog #:48-4801, BM8, eBioscience), APC rat anti-mouse CD11b (Catalog #:17-0112, M1/70, eBioscience), APC-Cy7 rat anti-mouse Ly-6G (Catalog #:127624, Biolegend), and BV711 rat anti-mouse Ly6c (Catalog #:128037, Biolegend). Cells were washed with 1% BSA, spun at 1300 rpm (330 X g), and fixed with 2% PFA at room temperature for 30 minutes. Cells were washed with 1% BSA, spun at 1300 rpm (330 X g), and resuspended in 1X PBS. After immunostaining, cells were analyzed on a BD LSRII flow cytometer. Data analysis was performed using FlowJo v10.8.1 software.

### RNA isolation and qRT-PCR

For qRT-PCR experiments, isolated single cells were sorted into tubes containing TRIzol using a BD FACSAriaII. RNA was isolated, transcribed into cDNA, and qRT-PCR performed using TaqMan real-time PCR. The following probes were used: CCR2 (Mm00438270_m1) and HPRT (Mm00446968_m1).

### Isolation of cells for single cell sequencing

Cells were prepared as described in the flow cytometry section. After blocking, cells were spun and incubated with the following antibodies in 1% BSA for 30 minutes: BV786 rat anti-mouse CD45 (Catalog #: 557659, 30-F11, BD Pharmigen), eFluor^®^450 rat anti-mouse F4/80 (Catalog #:48-4801, BM8, eBioscience), APC rat anti-mouse CD11b (Catalog #:17-0112, M1/70, eBioscience), APC-Cy7 rat anti-mouse Gr-1 (Catalog #:557661, RB6-8C5, BD Pharmingen), and Fixable Aqua Dead Cell Stain (catalog no. L34957; Invitrogen). After staining for 30 minutes, cells were spun, washed with 1% BSA, and sorted using a Becton–Dickenson FACSAriaII. We sorted ~25,000 live CD11b^+^ and F4/80^+^ cells from individual animals (n=2 per group) into individual BSA coated tubes. Cells were counted and approximately 5,000 cells from each animal were subjected to 10X genomics. This was done for n=2 animals from each experimental group; all animals were female.

### 10X genomics

10xChromium single cell libraries were prepared according to the standard protocol outlined in the manual. Briefly, sorted single cell suspension, 10x barcoded gel beads, and oil were loaded into Chromium™ Single Cell A Chip to capture single cells in nanoliter-scale oil droplets by Chromium™ Controller and to generate Gel Bead-In-Emulsions (GEMs). Full length cDNA libraries were prepared by incubation of GEMs in a thermocycler machine. GEMs containing cDNAs were broken and all single cell cDNA libraries were pooled together, cleaned using DynaBeads MyOne™ Silane beads (Fisher PN 37002D), and pre-amplified by PCR to generate sufficient mass for sequencing library construction. Sequencing libraries were constructed by following the steps: cDNA fragmentation, end repair & A-tailing, size selection by SPRIselect beads (Beckman Coulter, PN B23318), adaptor ligation, sample index PCR amplification, and a repeat of SPRIselect beads size selection. The final constructed single cell libraries were sequenced by Illumina Nextseq machine with total reads per cell targeted for a minimum of 50,000.

### Single cell sequencing data processing

For 10X Genomics single cells, the 10X Genomics Cellranger software (version 6.1.2), ‘mkfastq’, was used to create the fastq files from the sequencer. Following fastq file generation, Cellranger ‘count’ was used to align the raw sequence reads to the reference genome (mm10) using STAR. We also used –include-introns– so that downstream RNA velocity could be performed on spliced and unspliced transcripts. The ‘count’ software created 3 data files (barcodes.tsv, genes.tsv, matrix.mtx) from the ‘filtered_gene_bc_matrices’ folder that were loaded into the R package Seurat version 3.2 (58), which allows for selection and filtration of cells based on QC metrics, data normalization and scaling, and detection of highly variable genes. We followed the Seurat vignette (https://satijalab.org/seurat/pbmc3k_tutorial.html) to create the Seurat data matrix object. We then combined Seurat objects from each individual experiment using the ‘merge’ function. Following merging, we processed data to remove low quality cells by keeping all genes expressed in greater than 3 cells and cells with at least 200 detected copies. Cells with mitochondrial gene percentages over 5% and unique gene counts greater than 2,500 or fewer than 200 were discarded. The data were normalized using Seurat’s ‘NormalizeData’ function, which uses a global-scaling normalization method, LogNormalize, to normalize the gene expression measurements for each cell to the total gene expression. The result is multiplied by a scale factor of 1e4 and the result is log-transformed. Highly variable genes were then identified using the function ‘FindVariableGenes’ in Seurat. Genes were placed into 20 bins based on their average expression and removed using 0.0125 low cutoff, 3 high cutoff and a z-score cutoff of 0.5. We also regressed out the variation arising from library size and percentage of mitochondrial genes using the function ‘ScaleData’ in Seurat. We performed principal component analysis (PCA) of the variable genes as input and determined significant PCs based on the ‘JackStraw’ function in Seurat. The first 10 PCs were selected as input for Uniform Manifold Approximation and Projection (UMAP) dimension Reduction using the functions ‘FindClusters’ and ‘DimPlot’ in Seurat. To identify differentially expressed genes in each cell cluster, we used the function ‘FindAllMarkers’ in Seurat on the normalized gene expression data.

### Parabiosis

The parabiosis protocol was modified from the procedure published by Kamran and colleagues(59). For this procedure, incisions were made through the skin and muscle layer starting from the elbow joint and extending down the flank to the knee joint. Non-absorbable 3-0 interrupted sutures were placed around the knee and elbow joints to prevent strain along the suture lines, with care paid not to obstruct blood flow to the distal extremities. Analgesia was maintained on all mice according to IACUC guidelines. For these experiments, we used eight to 10-week-old female *Cx3cr1* ^gfp/gfp^ and CD45.1 mice (all mice were female). Mice were conjoined for six weeks, a time point that allows efficient exchange of immune cells between mice(49). After 6 weeks, mice were administered anesthesia and separated for individual analysis. Single cells were analyzed by flow cytometry as described above.

### Cystic mouse model

We induced cilia loss in six to ten-week-old conditional *Ift88* mice (CM mice) by injecting mice with a single intraperitoneal (IP) injection of tamoxifen at 6 mg/40 g body weight once daily for 3 consecutive days. Deletion of *Ift88* was confirmed by PCR using the following primers: 5`GCCTCCTGTTTCTTGACAACAGTG, GGTCCTAACAAGTAAGCCCAGTGTT3`, 5`CTGCACCAGCCATTTCCTCTAAGTCATGTA-3`.

### Unilateral renal ischemia-reperfusion injury (IR)

Three weeks after tamoxifen induction, control or CM mice were subjected to unilateral ischemia reperfusion injury by clamping the left renal pedicle for 30 minutes followed by reperfusion. Mice were injected with buprenorphine (0.05 mg/kg) for pain relief and allowed to recover on a 37°C heating pad. Mice were sacrificed 8 weeks post injury and cystic index calculated as described in the fixation and tissue processing section below.

### Fixation and tissue processing

Following harvesting, mouse kidneys were immersion fixed in 4% (wt/vol) paraformaldehyde overnight at 4°C, switched to 70% ethanol overnight, embedded in paraffin, sectioned at 5 µm, and stained using hematoxylin and eosin (H&E). Cystic index was quantified using Image J software and was calculated by determining the total cystic area divided by the total kidney area.

### Pseudotime analysis

For pseudotime analysis, we used Monocle 2 (http://cole-trapnell-lab.github.io/monocle-release/docs/#constructing-single-cell-trajectories) or RNA velocity software (https://scvelo.readthedocs.io/) and followed the standard vignette for processing single cell data.

### Pathway analysis

The Seurat function ‘FindAllMarkers’ was run to identify differentially expressed genes that distinguished one cluster from others as described in the text. A list of genes enriched in each cluster is shown in **Supplemental File 1**. Pathway analysis was performed on genes enriched in each cluster of cells (adjusted p value > 0.05) using Metascape software(60). The top 5-6 pathways that were enriched in each cluster of cells is shown.

### Data availability

Raw single cell RNA sequencing data can be downloaded using the following GEO number: GSE193892.

### Code availability

All code used for data analysis and visualization are included as **Supplemental File 2**.

### Statistics and data analysis

Data were presented as mean ± SEM. ANOVA and Student T tests were used for statistical analysis, and differences were considered significant for P values less than 0.05.

## Data availability statement

The datasets presented in this study can be found in online repositories. The names of the repository/repositories and accession number(s) can be found in the article/**Supplementary Material**.

## Ethics statement

The animal study was reviewed and approved by Institutional Animal Care and Use Committee (IACUC) at the University of Alabama at Burmingham (UAB) and University of Oklahoma Health Sciences Center (OUHSC).

## Author contributions

AY, SB, and KZ designed the research studies. AY, SB, CS, UA, RS, ID, AC, MS, JL, ZL, EA, SK, BM, SL, MC, KJ, LZ, and KZ conducted experiments and acquired data. AY, SB, CS, UA, RS, JL, ZL, NMG, DZR, EA, KJ, JG, DC, SS, MH, FG, and KZ analyzed data. JG, MM, DC, MH, FG, and KZ provided reagents. AY and KZ wrote the manuscript. All authors contributed to the article and approved the submitted version.

## Supplementary material

Supplementary Figure 1 Analysis of monocytes and tissue resident macrophages in the kidney. **(A)** Gating strategy used to sort CD11b and F4/80 double positive cells in the kidney. **(B)** Feature plots of key immune cell markers in clusters from. **(C)** Feature plot showing expression of the mouse neutrophil marker *S100a8.*

Supplementary Figure 2 Traditional gating strategy used to identify monocytes, KRM, and dendritic cells (DCs) in the kidney in both flow cytometry and scRNAseq data. **(A)** Representative FACS plots showing *Ccr2* reporter activity (*Ccr2*-GFP) and CCR2 antibody positive cells in KRM isolated from 6-8-week-old wild type mice. **(B)** Quantification of the number of CCR2 antibody positive KRM in *Ccr2* control or knockout (*Ccr2* ^gfp/gfp^) mice at 6 to 8 weeks of age. T-test. **(C)** FACS plots showing the classical gating strategy used to identify monocytes, KRM, and DCs in the kidney. **(D)** scRNAseq UMAPs showing the classical gating strategy used to identify monocytes, KRM, and DCs in the kidney.

Supplementary Figure 3 New gating strategy used to identify monocytes, KRM, and dendritic cells (DCs) in the kidney in both flow cytometry and scRNAseq data. **(A)** FACS plots showing how we identified IM and KRM for further analysis in panel B. **(B)** FACS plots showing the new gating strategy used to identify monocyte, KRM, and DC heterogeneity in the kidney. For IM and KRM, an FMO control is included for each cell population. **(C)** scRNAseq UMAPs showing the IM and KRM marker genes that were used to identify cellular heterogeneity.

Supplementary Figure 4 Gating strategy used to identify IM heterogeneity in the blood. **(A)** FACS plots showing the gating strategy used to identify blood monocytes. **(B)** FACS plots showing staining for UPAR, LRP1, and SCA-1 in Ly6c^hi^ blood monocytes. **(C)** Quantification of blood Ly6c^hi^ monocyte subsets as a fraction of all blood Ly6c^hi^ monocytes. SCA-1^+^ monocytes were not detected (ND).

Supplementary Figure 5 Characteristics of *Ccr2^+^* KRM. **(A)** Expression of *Cx3cr1*-GFP in KRM and IMs isolated from the kidney of *Ccr2* ^rfp/wt^, *Cx3cr1* ^gfp/wt^ mice at 6-8 weeks of age. **(B)** Quantification of the *Ccr2^+^* KRM abundance in scRNAseq or flow cytometry (both Clec12a+ or *Ccr2-*RFP^+^ approaches). **(C)** qRT-PCR data showing the level of expression of *Ccr2* in flow sorted infiltrating monocytes (IMs), *Ccr2^-^* KRM, and *Ccr2^+^* KRM. N=5 mice. **(D,E)** Metascape pathway analysis of genes that were significantly enriched (adjusted p value < 0.05) in **(D)** KRM or **(E)** IM subsets in the kidney. For this analysis, a list of genes enriched in each cluster was generated by doing a head-to-head comparison of KRM or IM subsets, respectively. **(F)** Representative images of the kidney cortex and medulla of *Ccr2* ^rfp/wt^ *Cx3cr1* ^gfp/wt^ mice stained with the pan macrophage marker F4/80. A zoomed in version is shown in **Figure 1G**.

Supplementary Figure 6 Loss of *Ccr2* only impacts *Ccr2+* KRM numbers in aged animals. **(A,B)** Quantification of Ly6c^hi^ monocyte number in the **(A)** blood or **(B)** kidney of 6-8 week old control (*Ccr2* ^rfp/wt^) or *Ccr2* knockout (*Ccr2* ^rfp/rfp^) mice. T-test. **(C)** Representative FACS plots showing Ccr2-RFP expression in KRM isolated from *Ccr2* ^rfp/wt^ or *Ccr2* ^rfp/rfp^ mice at 6-8 weeks of age. **(D)** Quantification of *Ccr2^+^* KRM number in *Ccr2* ^rfp/wt^ or *Ccr2* ^rfp/rfp^ mice at 6-8 weeks of age. T-test. **(E)** Quantification of *Ccr2^+^* KRM number in *Ccr2* ^rfp/wt^ or *Ccr2* ^rfp/rfp^ mice at 6 months of age. T-test. *P< 0.05

Supplementary Figure 7 Analysis of monocytes and macrophages in the *Cx3cr1* ^gfp/gfp^ mice. **(A)** Feature plots showing expression of key IM and KRM genes in cells from **Figure 3**.

Supplementary Figure 8 Analysis of IM and KRM subsets in control and *Cx3cr1* ^gfp/gfp^ mice. **(A)** FACS plots showing expression of UPAR, LRP1, or SCA-1 in IMs *via* flow cytometry. Mice were harvested at 6-8 weeks of age. **(B,C)** Quantification of flow cytometry data analyzing IM subsets as **(B)** a percentage of CD11b and F4/80 positive cells or **(C)** as a percentage of live single cells in the kidney. Two-way ANOVA. **(D)** FACS plots showing expression of CD63, Clec12a, and CD206 in KRM *via* flow cytometry. Mice were harvested at 6-8 weeks of age. Quantification of the data is shown in . **(E)** RNA velocity analysis on the composite UMAP shown in . **(F)** Plot showing expression of *Cx3cl1* in different segments of the nephron. Data were obtained using the Kidney Cell Explorer (45).

Supplementary Figure 9 Loss of *Cx3cr1* does not result in a preferential accumulation of CD45.1 or CD45.2 monocytes, neutrophils, or T cells in the blood of CD45.2 *Cx3cr1* ^gfp/gfp^ mice. **(A)** Quantification of chimerism in blood Ly6c^hi^ monocytes isolated from CD45.1 WT and CD45.2 *Cx3cr1* ^gfp/gfp^ mice 6 weeks post hook-up. T-test. **(B)** Quantification of CD45.1 and CD45.2 monocyte, neutrophil, and T cell numbers in blood isolated from CD45.1 WT or CD45.2 *Cx3cr1* ^gfp/gfp^ mice. Two-way ANOVA. **(C)** Quantification of the number of blood Ly6c^hi^ monocytes in control (*Cx3cr1* ^gfp/wt^) or *Cx3cr1* knockout (*Cx3cr1* ^gfp/gfp^) mice at 6-8 weeks of age. Two-way ANOVA.
