# Supplementary figures and images for: Cx3cr1 controls kidney resident macrophage heterogeneity
Source: Front Immunol. 2023 May 15;14:1082078. doi: 10.3389/fimmu.2023.1082078 (PMC10225589; doi:10.3389/fimmu.2023.1082078)

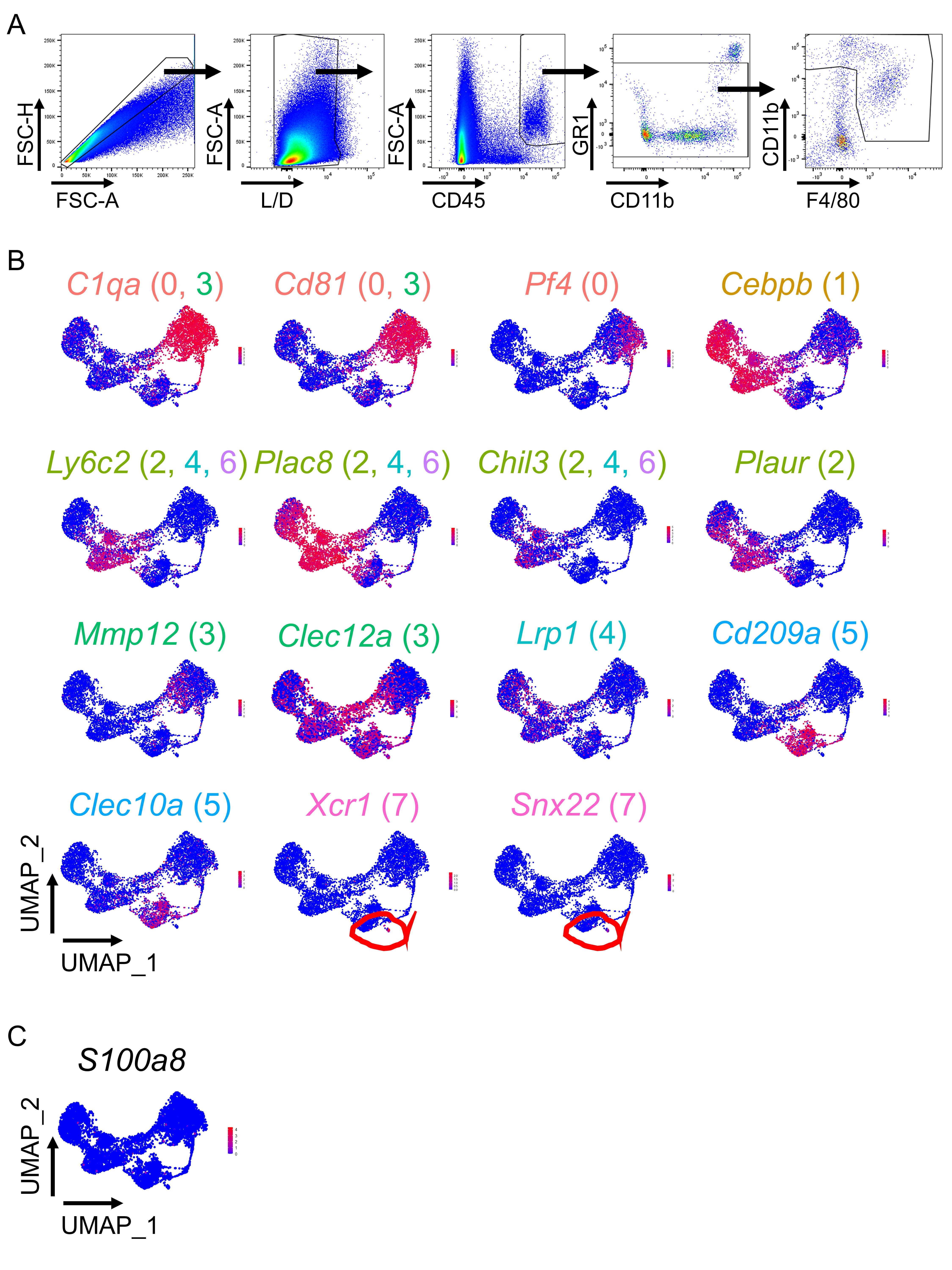

Supplement: Supplementary Figure 1 — Analysis of monocytes and tissue resident macrophages in the kidney. (A) Gating strategy used to sort CD11b and F4/80 double positive cells in the kidney. (B) Feature plots of key immune cell markers in clusters from. (C) Feature plot showing expression of the mouse neutrophil marker S100a8. [file Image_1.jpeg]

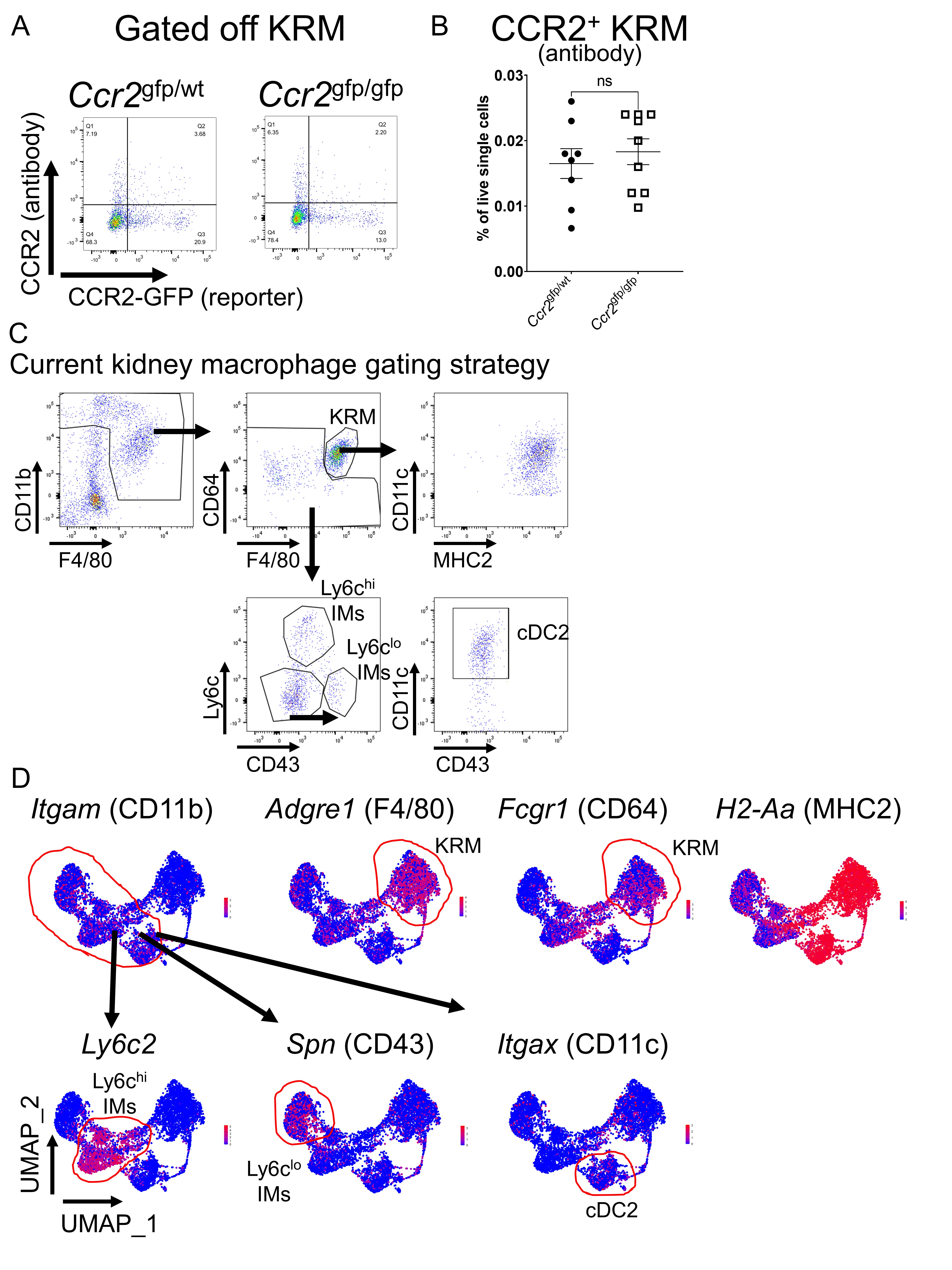

Supplement: Supplementary Figure 2 — Traditional gating strategy used to identify monocytes, KRM, and dendritic cells (DCs) in the kidney in both flow cytometry and scRNAseq data. (A) Representative FACS plots showing Ccr2 reporter activity (Ccr2-GFP) and CCR2 antibody positive cells in KRM isolated from 6-8-week-old wild type mice. (B) Quantification of the number of CCR2 antibody positive KRM in Ccr2 control or knockout (Ccr2 gfp/gfp) mice at 6 to 8 weeks of age. T-test. (C) FACS plots showing the classical gating strategy used to identify monocytes, KRM, and DCs in the kidney. (D) scRNAseq UMAPs showing the classical gating strategy used to identify monocytes, KRM, and DCs in the kidney. [file Image_2.jpeg]

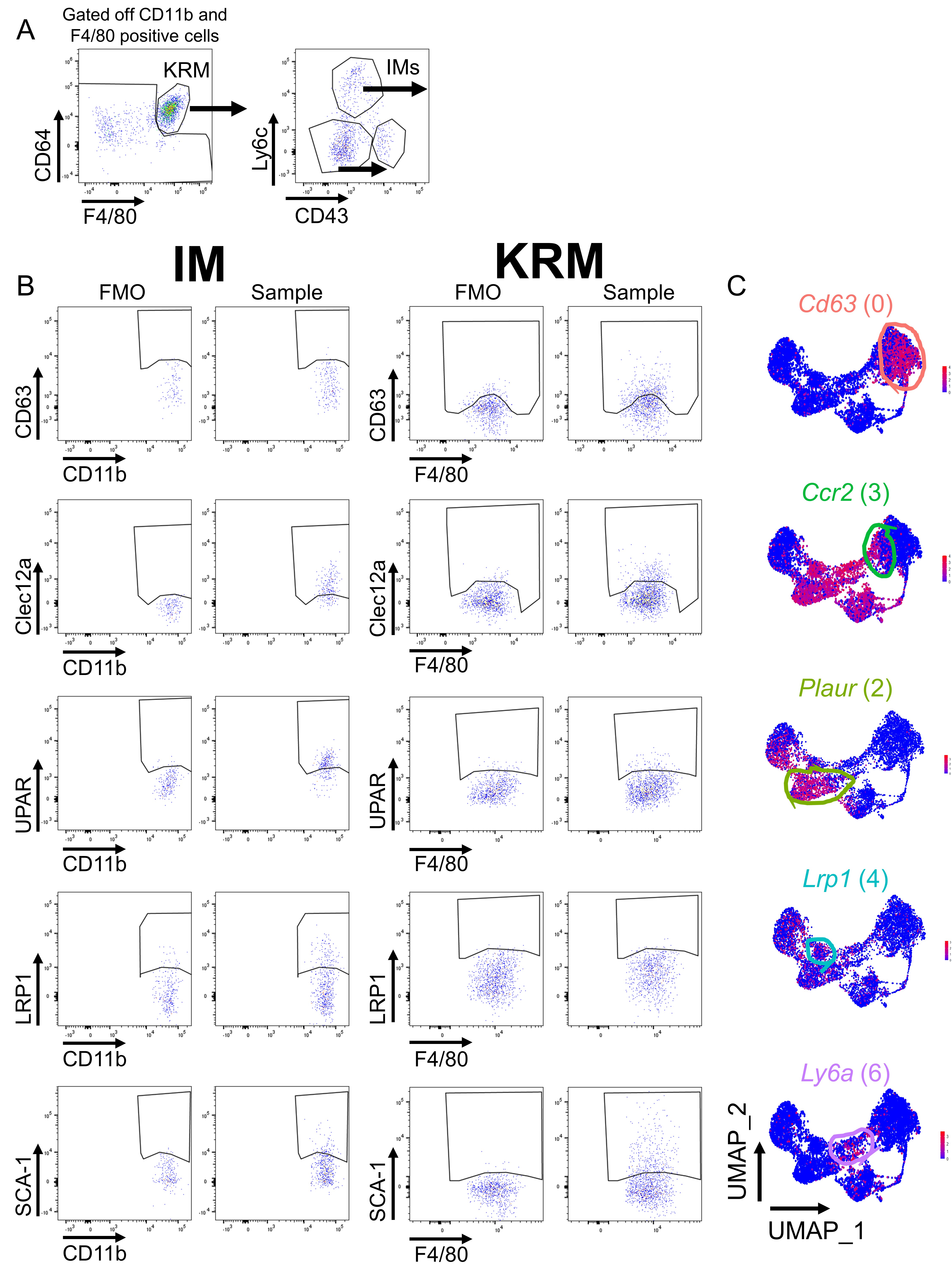

Supplement: Supplementary Figure 3 — New gating strategy used to identify monocytes, KRM, and dendritic cells (DCs) in the kidney in both flow cytometry and scRNAseq data. (A) FACS plots showing how we identified IM and KRM for further analysis in panel B. (B) FACS plots showing the new gating strategy used to identify monocyte, KRM, and DC heterogeneity in the kidney. For IM and KRM, an FMO control is included for each cell population. (C) scRNAseq UMAPs showing the IM and KRM marker genes that were used to identify cellular heterogeneity. [file Image_3.jpeg]

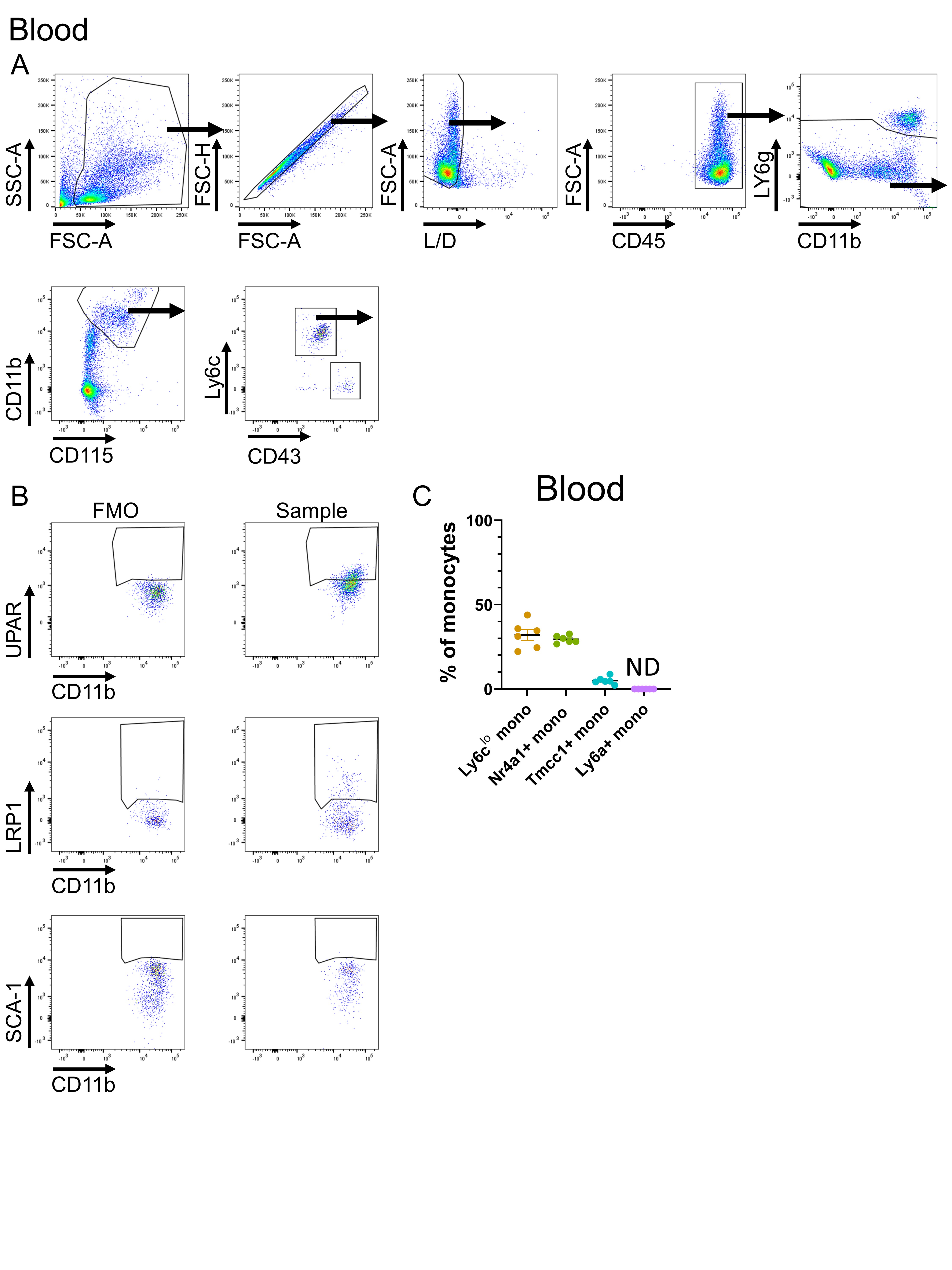

Supplement: Supplementary Figure 4 — Gating strategy used to identify IM heterogeneity in the blood. (A) FACS plots showing the gating strategy used to identify blood monocytes. (B) FACS plots showing staining for UPAR, LRP1, and SCA-1 in Ly6chi blood monocytes. (C) Quantification of blood Ly6chi monocyte subsets as a fraction of all blood Ly6chi monocytes. SCA-1+ monocytes were not detected (ND). [file Image_4.jpeg]

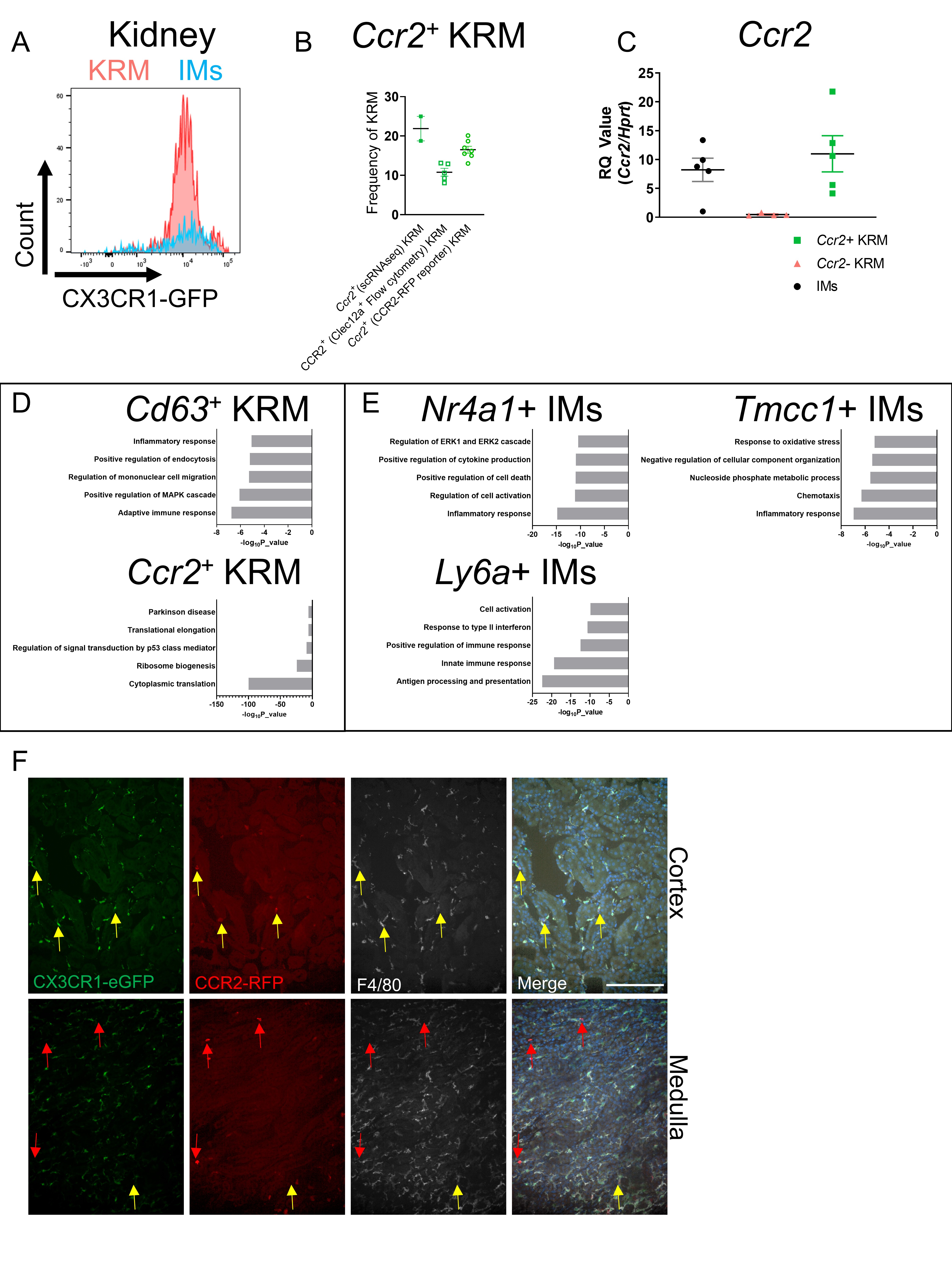

Supplement: Supplementary Figure 5 — Characteristics of Ccr2+ KRM. (A) Expression of Cx3cr1-GFP in KRM and IMs isolated from the kidney of Ccr2 rfp/wt, Cx3cr1 gfp/wt mice at 6-8 weeks of age. (B) Quantification of the Ccr2+ KRM abundance in scRNAseq or flow cytometry (both Clec12a+ or Ccr2-RFP+ approaches). (C) qRT-PCR data showing the level of expression of Ccr2 in flow sorted infiltrating monocytes (IMs), Ccr2- KRM, and Ccr2+ KRM. N=5 mice. (D,E) Metascape pathway analysis of genes that were significantly enriched (adjusted p value < 0.05) in (D) KRM or (E) IM subsets in the kidney. For this analysis, a list of genes enriched in each cluster was generated by doing a head-to-head comparison of KRM or IM subsets, respectively. (F) Representative images of the kidney cortex and medulla of Ccr2 rfp/wt Cx3cr1 gfp/wt mice stained with the pan macrophage marker F4/80. A zoomed in version is shown in Figure 1G. [file Image_5.jpeg]

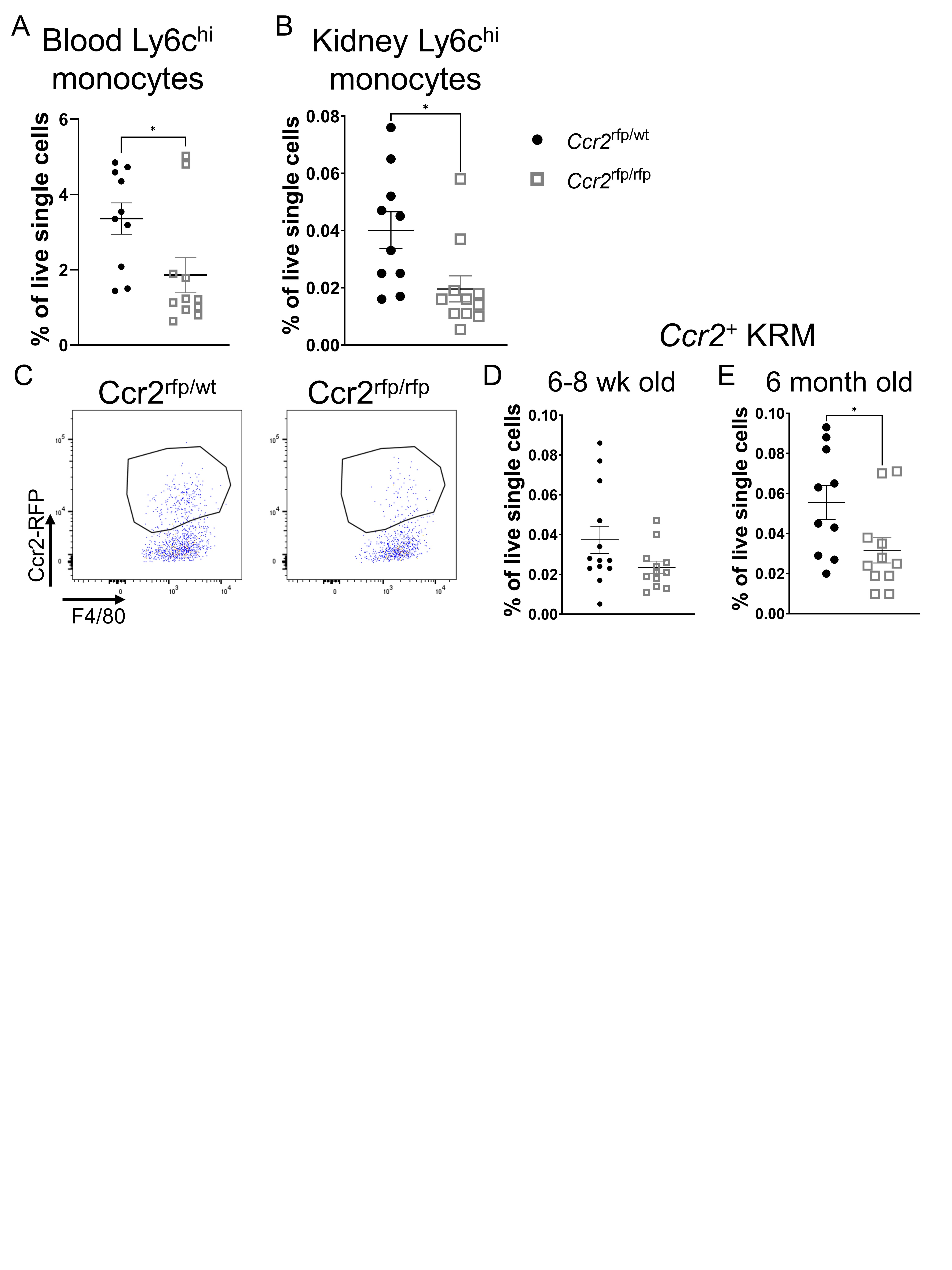

Supplement: Supplementary Figure 6 — Loss of Ccr2 only impacts Ccr2+ KRM numbers in aged animals. (A,B) Quantification of Ly6chi monocyte number in the (A) blood or (B) kidney of 6-8 week old control (Ccr2 rfp/wt) or Ccr2 knockout (Ccr2 rfp/rfp) mice. T-test. (C) Representative FACS plots showing Ccr2-RFP expression in KRM isolated from Ccr2 rfp/wt or Ccr2 rfp/rfp mice at 6-8 weeks of age. (D) Quantification of Ccr2+ KRM number in Ccr2 rfp/wt or Ccr2 rfp/rfp mice at 6-8 weeks of age. T-test. (E) Quantification of Ccr2+ KRM number in Ccr2 rfp/wt or Ccr2 rfp/rfp mice at 6 months of age. T-test. *P< 0.05 [file Image_6.jpeg]

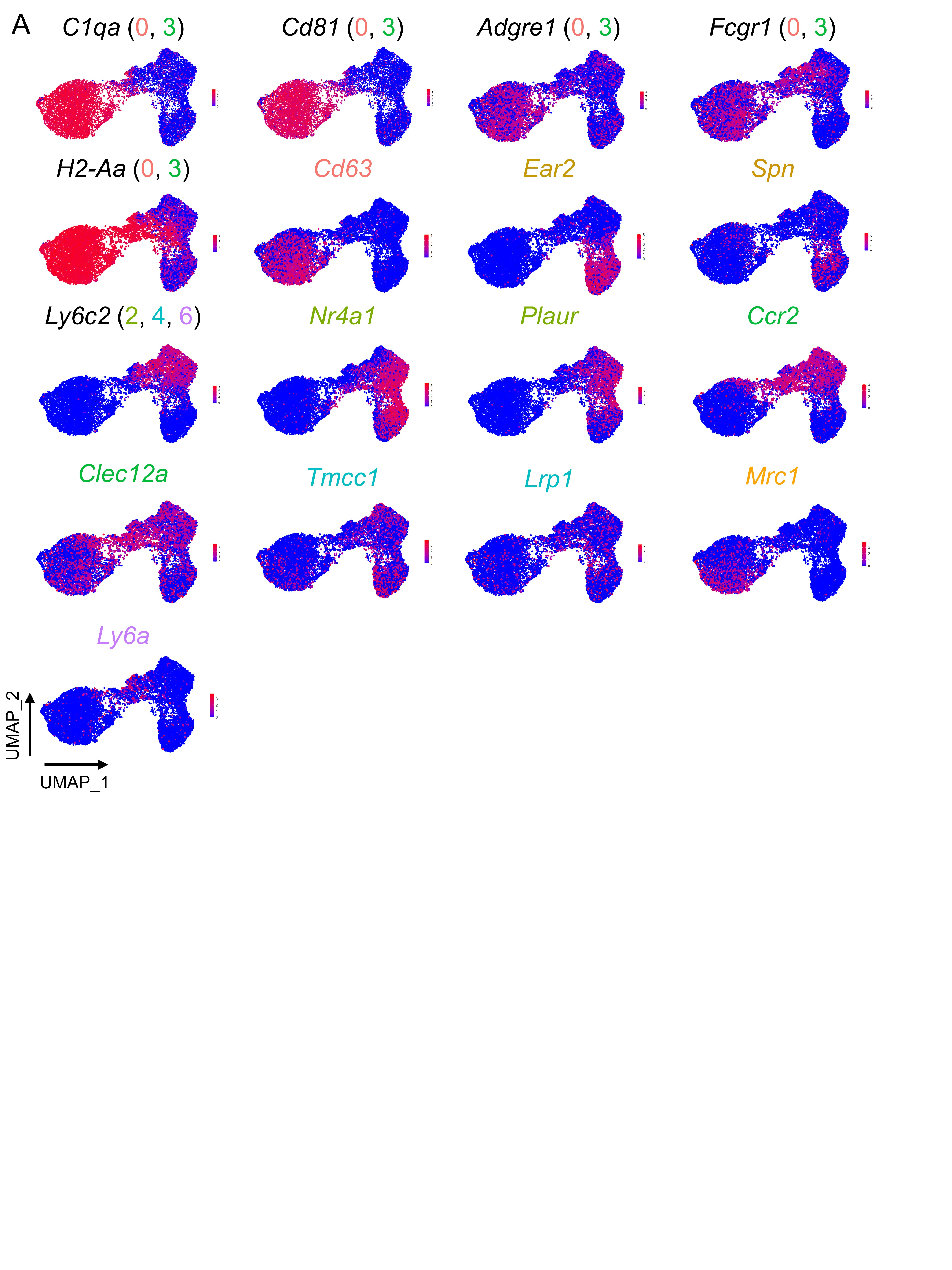

Supplement: Supplementary Figure 7 — Analysis of monocytes and macrophages in the Cx3cr1 gfp/gfp mice. (A) Feature plots showing expression of key IM and KRM genes in cells from Figure 3. [file Image_7.jpeg]

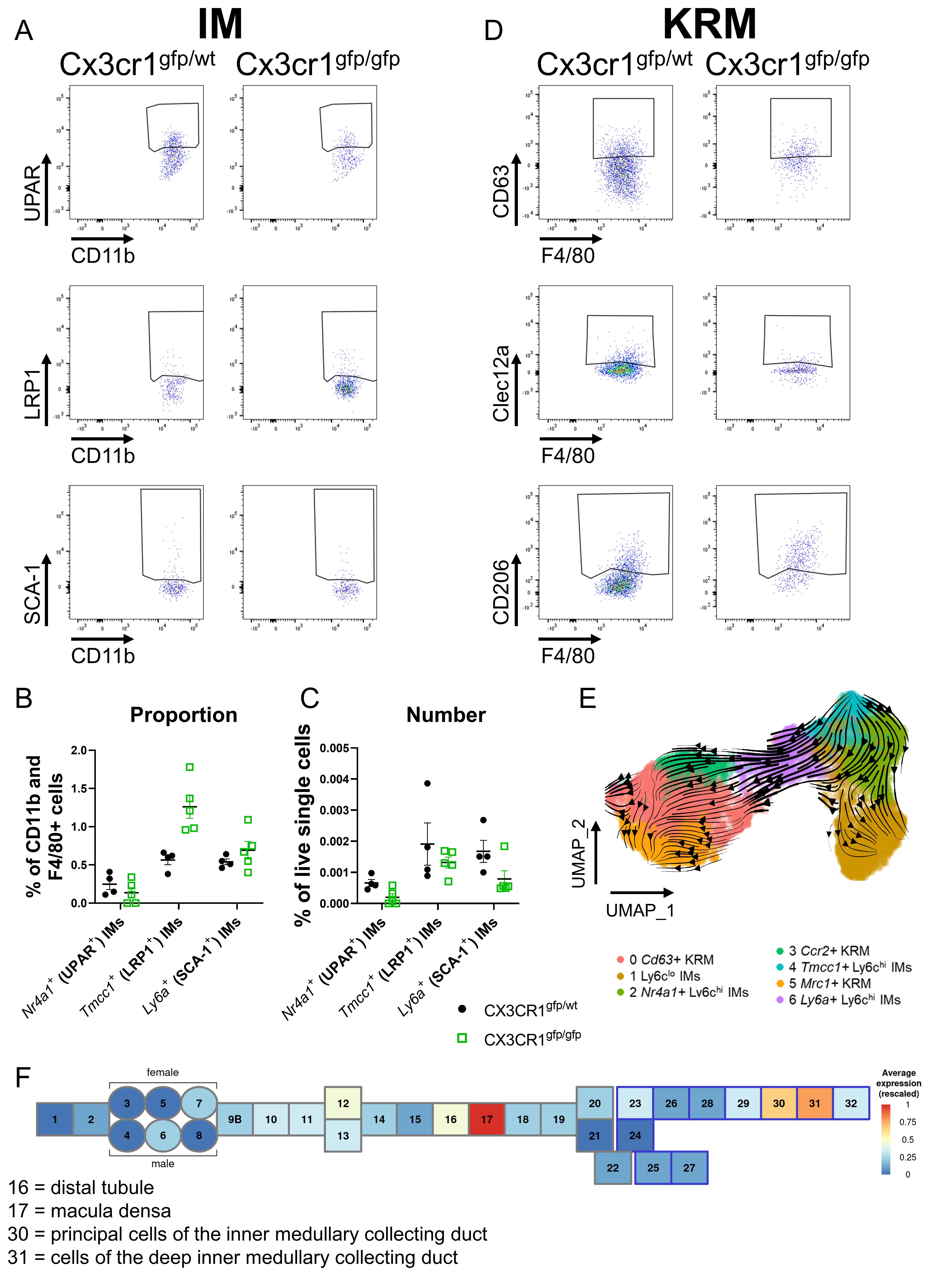

Supplement: Supplementary Figure 8 — Analysis of IM and KRM subsets in control and Cx3cr1 gfp/gfp mice. (A) FACS plots showing expression of UPAR, LRP1, or SCA-1 in IMs via flow cytometry. Mice were harvested at 6-8 weeks of age. (B,C) Quantification of flow cytometry data analyzing IM subsets as (B) a percentage of CD11b and F4/80 positive cells or (C) as a percentage of live single cells in the kidney. Two-way ANOVA. (D) FACS plots showing expression of CD63, Clec12a, and CD206 in KRM via flow cytometry. Mice were harvested at 6-8 weeks of age. Quantification of the data is shown in . (E) RNA velocity analysis on the composite UMAP shown in . (F) Plot showing expression of Cx3cl1 in different segments of the nephron. Data were obtained using the Kidney Cell Explorer (45). [file Image_8.jpeg]

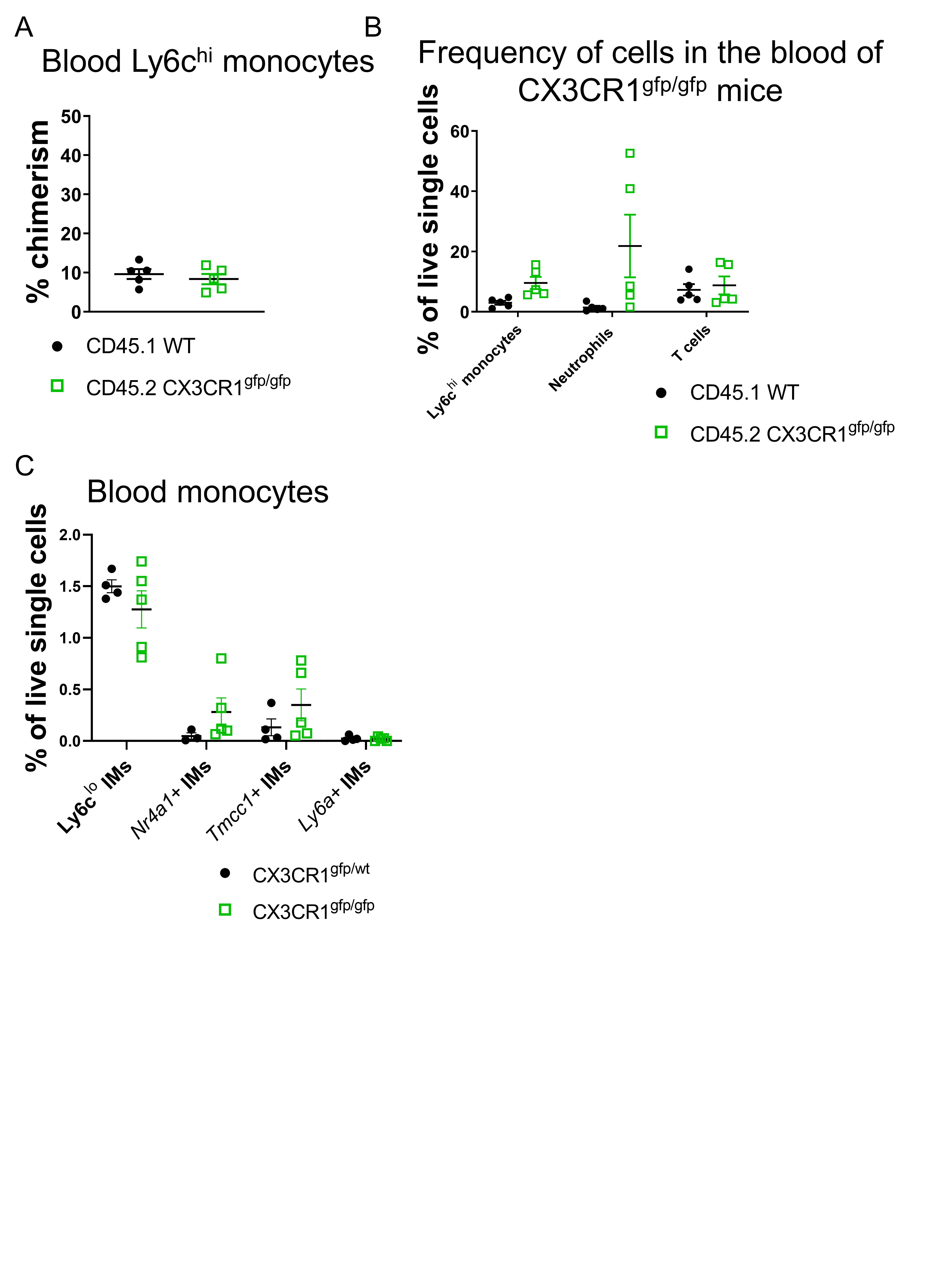

Supplement: Supplementary Figure 9 — Loss of Cx3cr1 does not result in a preferential accumulation of CD45.1 or CD45.2 monocytes, neutrophils, or T cells in the blood of CD45.2 Cx3cr1 gfp/gfp mice. (A) Quantification of chimerism in blood Ly6chi monocytes isolated from CD45.1 WT and CD45.2 Cx3cr1 gfp/gfp mice 6 weeks post hook-up. T-test. (B) Quantification of CD45.1 and CD45.2 monocyte, neutrophil, and T cell numbers in blood isolated from CD45.1 WT or CD45.2 Cx3cr1 gfp/gfp mice. Two-way ANOVA. (C) Quantification of the number of blood Ly6chi monocytes in control (Cx3cr1 gfp/wt) or Cx3cr1 knockout (Cx3cr1 gfp/gfp) mice at 6-8 weeks of age. Two-way ANOVA. [file Image_9.jpeg]
