# Supplementary material for: Cx3cr1 controls kidney resident macrophage heterogeneity
Source: Front Immunol. 2023 May 15;14:1082078. doi: 10.3389/fimmu.2023.1082078 (PMC10225589; doi:10.3389/fimmu.2023.1082078)
Supplement: Supplementary file 11 [file DataSheet_2.docx]

**Summary of scripts used for manuscript:**

**Seurat:**

#Load libraries

>library(dplyr)

>library(Seurat)

#Load data:

>data.file <- Read10X("filtered_feature_bc_matrix/")

>sample1 <- CreateSeuratObject(counts = data.file, project = "project_1", min.cells = 3, min.features = 200)

#Analyze mitochondria

> **sample1**[["percent.mt"]] <- PercentageFeatureSet(**sample1**, pattern = "^mt-")

> VlnPlot(**sample1**, features = c("nFeature_RNA", "nCount_RNA", "percent.mt"), ncol = 3)

> plot1 <- FeatureScatter(**sample1**, feature1 = "nCount_RNA", feature2 = "percent.mt")

> plot2 <- FeatureScatter(**sample1**, feature1 = "nCount_RNA", feature2 = "nFeature_RNA")

> CombinePlots(plots = list(plot1, plot2))

> **sample1** <- subset(**sample1**, subset = nFeature_RNA > 200 & nFeature_RNA < 3000 & percent.mt < 5)

#Normalize the data

> **sample1** <- NormalizeData(**sample1**, normalization.method = "LogNormalize", scale.factor = 10000)

> **sample1** <- FindVariableFeatures(**sample1**, selection.method = "vst", nfeatures = 2000)

> top10 <- head(VariableFeatures(**sample1**), 10)

> plot1 <- VariableFeaturePlot(**sample1**)

> plot2 <- LabelPoints(plot = plot1, points = top10, repel = TRUE)

> CombinePlots(plots = list(plot1, plot2))

#Scaling the data

> all.genes <- rownames(**sample1**)

> **sample1** <- ScaleData(**sample1**, features = all.genes)

#Performing linear dimensional reduction

> **sample1** <- RunPCA(**sample1**, features = VariableFeatures(object = **sample1**))

> VizDimLoadings(**sample1**, dims = 1:2, reduction = "pca")

> DimPlot(**sample1**, reduction = "pca")

> DimHeatmap(**sample1**, dims = 1, cells = 500, balanced = TRUE)

> DimHeatmap(**sample1**, dims = 1:15, cells = 500, balanced = TRUE)

#Determining the dimensionality of the data set

> **sample1** <- JackStraw(**sample1**, num.replicate = 100)

> **sample1** <- ScoreJackStraw(**sample1**, dims = 1:20)

> JackStrawPlot(**sample1**, dims = 1:15)

#Cluster the cells

> **sample1** <- FindNeighbors(**sample1**, dims = 1:10)

> **sample1** <- FindClusters(**sample1**, resolution = 0.3)

* note used 0.3 as it lead to most defined clustering

#Run non-linear dim reductinos

> **sample1** <- RunUMAP(**sample1**, dims = 1:10)

> DimPlot(**sample1**, reduction = "umap", pt.size = 3)

#Save the files

> saveRDS(**sample1**, file = "**sample1**.rds")

#Find markers that uniquely identifies each cluster

> **sample1**.markers <- FindAllMarkers(**sample1**, only.pos = TRUE, min.pct = 0.25, logfc.threshold = 0.25)

> **sample1**.markers %>% group_by(cluster) %>% top_n(n = 2, wt = avg_logFC)

#Find markers that distinguishes one cluster from another cluster

> cluster4.markers <- FindMarkers(**sample1**, ident.1 = 4, ident.2 = c(0), min.pct = 0.25)

> head(cluster4.markers, n = 10)

#Identify and plot (heatmap) top 5 markers of each cluster

> top5 <- **sample1**.markers %>% group_by(cluster) %>% top_n(n = 5, wt = avg_logFC)

> DoHeatmap(**sample1**, features = top5$gene) + NoLegend()

**Monocle 2:**

Note: “3” is whatever cluster you want pseudotime to start with

#Load libraries

| > library(DDRTree)  > library(devtools)  > library(monocle)  > library(pheatmap)  #CreateCDS from Seurat object “sample1”  > sample1 <- readRDS("~/sample1.rds")  > data <- as(as.matrix(sample1@assays$RNA@data), 'sparseMatrix')  > pd <- new('AnnotatedDataFrame', data = sample1@meta.data)  > fData <- data.frame(gene_short_name = row.names(data), row.names = row.names(data))  > fd <- new('AnnotatedDataFrame', data = fData)  > cds <- newCellDataSet(data, phenoData = pd, featureData = fd, lowerDetectionLimit = 0.5, expressionFamily = negbinomial.size())  #Estimate size factors and dispersions  > cds <- estimateSizeFactors(cds)  > cds <- estimateDispersions(cds)  #Filter low quality cells  > pData(cds)$Total_mRNAs <- Matrix::colSums(exprs(cds))  > cds <- cds[,pData(cds)$Total_mRNAs < 1e6]  > upper_bound <- 10^(mean(log10(pData(cds)$Total_mRNAs)) +  2*sd(log10(pData(cds)$Total_mRNAs)))  > lower_bound <- 10^(mean(log10(pData(cds)$Total_mRNAs)) -  2*sd(log10(pData(cds)$Total_mRNAs)))  > qplot(Total_mRNAs, data = pData(cds), color = seurat_clusters, geom =  "density") +  geom_vline(xintercept = lower_bound) +  geom_vline(xintercept = upper_bound)  > cds <- cds[,pData(cds)$Total_mRNAs > lower_bound &  pData(cds)$Total_mRNAs < upper_bound]  > cds <- detectGenes(cds, min_expr = 0.1)  > print(head(fData(cds)))  #Cluster cells  > expressed_genes <- row.names(subset(fData(cds), num_cells_expressed >= 10))  > disp_table <- dispersionTable(cds)  > unsup_clustering_genes <- subset(disp_table, mean_expression >= 0.1)  > cds <- setOrderingFilter(cds, unsup_clustering_genes$gene_id)  > plot_ordering_genes(cds)  > cds <- reduceDimension(cds, max_components = 2, num_dim = 6,  reduction_method = 'tSNE', verbose = T)  > cds <- clusterCells(cds, num_clusters = 2)  > plot_cell_clusters(cds)  > cds <- setOrderingFilter(cds, unsup_clustering_genes)  > plot_ordering_genes(cds)  > cds <- reduceDimension(cds, max_components = 2, method = 'DDRTree')  > cds <- orderCells(cds)  > plot_cell_trajectory(cds, color_by = "Cluster")  > plot_cell_trajectory(cds, color_by = "seurat_clusters") |
| --- |
| #Assigning time “0” of pseudotime  > plot_cell_trajectory(cds, color_by = "State")  > GM_state <- function(cds){  if (length(unique(pData(cds)$State)) > 1){  T0_counts <- table(pData(cds)$State, pData(cds)$seurat_clusters)[,"3"]  return(as.numeric(names(T0_counts)[which  (T0_counts == max(T0_counts))]))  } else {  return (1)  }  }  >  > cds <- orderCells(cds, root_state = GM_state(cds))  > plot_cell_trajectory(cds, color_by = "Pseudotime")  > plot_cell_trajectory(cds, color_by = "State", cell_size = 3) +  facet_wrap(~State, nrow = 1)  #Plotting genes in pseudotime  > blast_genes <- row.names(subset(fData(cds), gene_short_name %in% c("C1qa")))  > plot_genes_jitter(cds[blast_genes,],  grouping = "State",  min_expr = 0.1)  > unsup_clustering_genes <- row.names(subset(fData(cds),  num_cells_expressed >= 10))  > cds_filtered <- cds[expressed_genes,]  > my_genes <- row.names(subset(fData(cds_filtered),  gene_short_name %in% c("C1qa", "Fos")))  > cds_subset <- cds_filtered[my_genes,]  > plot_genes_in_pseudotime(cds_subset, color_by = "seurat_clusters") |
| \|  \| \| --- \| |

#Differential expression analysis- picking genes

> marker_genes <- row.names(subset(fData(cds),

gene_short_name %in% c("Cd63", "C1qa", "Ccr2",

"Apoe", "Sepp1","Pf4",

"Napsa", "Clec12a", "Fos",

"Junb", "Dusp1")))

> diff_test_res <- differentialGeneTest(cds[marker_genes,])

> sig_genes <- subset(diff_test_res, qval < 0.1)

> sig_genes[,c("gene_short_name", "pval", "qval")]

> MYOG_ID1 <- cds[row.names(subset(fData(cds), gene_short_name %in% c("C1qa", "Apoe"))),]

> plot_genes_jitter(MYOG_ID1, grouping = "seurat_clusters", ncol= 2)

#Analyzing branches in single cell trajectories

> BEAM_sample1 <- BEAM(cds, branch_point = 1, cores = 1)

> BEAM_sample1 <- BEAM_sample1[order(BEAM_sample1$qval),]

> BEAM_sample1 <- BEAM_sample1[,c("gene_short_name", "pval", "qval")]

> plot_genes_branched_heatmap(cds[row.names(subset(BEAM_sample1,

qval < 1e-4)),],

branch_point = 1,

num_clusters = 4,

cores = 1,

use_gene_short_name = T,

show_rownames = T)

> sample1_genes <- row.names(subset(fData(cds),

gene_short_name %in% c("C1qa", "Sepp1", "Ccr2")))

> plot_genes_branched_pseudotime(cds[sample1_genes,],

branch_point = 2,

color_by = "seurat_clusters",

ncol = 1)
